# Supplementary material for: Construction of disease-specific cytokine profiles by associating disease genes with immune responses
Source: PLoS Comput Biol. 2022 Apr 11;18(4):e1009497. doi: 10.1371/journal.pcbi.1009497 (PMC9022887; doi:10.1371/journal.pcbi.1009497)
Supplement: S1 Text — Method notes for embedding and grid searching. Fig A: Network sparsity within and between known functional modules in protein-protein interaction (PPI) networks. Fig B: The predicted Association Scores (Y-axis) of 8,521,944 edges correlate with their known STRING confidence scores. Fig C: The distribution of Association Scores. Fig D: Predicted Association Scores correlate with known confidence scores in STRING. Fig E: The histogram of the number of genes associated with each of the 171 diseases. Fig F: The NAAS distribution within each bin defined by the number of genes associated with a disease. Fig G: The Number of genes associated with diseases is plotted against the P-value estimating the correlation between the predicted NAAS and the literature sampling frequency of cytokines. Fig H: The NAAS between aneurysm and each of the 79 cytokines are plot against the literature sampling frequency in aneurysm. Fig I: Graph plots showing interactions between pathogenesis genes and inflammatory responses. Fig J: Hierarchical structure showing information flow from pathogenesis genes to inflammatory responses. Table A: Statistics of selected sets from STRING and three classes of functional modules. Table B: Gene signatures identified for the six groups of cytokines. Table C: The predicted cytokine profiles correlate with the known literature sampling frequency in ImmuneXpresso for the 171 well-studied diseases. Table D: The 171 diseases classified into three clusters based on their cytokine profiles. Table E: Disease-associated genes in the well-connected modules formed by pathogenesis genes, receptors, and essential cytokines identified by spectrum partition in the context of five immune disorders. Table F: Frequency (#) in the five diseases. (DOCX) [file pcbi.1009497.s001.docx]

Supplementary Material for the manuscript “Construction of Disease-specific Cytokine Profiles by Associating Disease Genes with Immune Responses”

**Method notes for embedding and grid searching**

To create a computational representation of the STRING network, we applied node2vec node embedding techniques to translate genes into vectors which capture their network locations. A total of 9,250,034 edges between the 14,707 human genes we considered were present in STRING. 728,090 of those edges had confidence scores above 800, and thus were used to train the embedding space. We evaluated the feature representations obtained through node2vec on a standard supervised learning task: unsupervised classification of higher-level network modules with random seeds. We employed a grid search to find and compare different hyperparameter settings and use the silhouette score as our evaluative performance metric. Our final hyperparameters were: length of walks=30, number of walks=10, min count=1, batch word=6, window=10. We calculated all 108,140,571 pairwise distances between the 14,707 genes. Of these, 8,521,944 pairs have STRING confidence scores ranging between 21-800 (the 728,090 pairs with scores above 800 are used for training). The pairs are novel.

Fig A. We inspected network sparsity within and between known functional modules in protein-protein interaction (PPI) networks. The connection rate is defined as the ratio of the number of actual links in the graph to the total number of possible links present in a complete graph. First, we found that the links within modules for immune response (Intra-ME) are under-represented compared to the average connection rate within metabolic and signaling modules (which are more heavily studied). The low connection rates within the immune response modules result from the lack of informative resources about immune response. Second, we found that the associations between functional modules are not well defined, suggesting that we need to better define the associations between immune response and functional modules.

Modules of ME are from <http://www.immprot.org/>. Modules of HM are from <http://www.gsea-msigdb.org/gsea/msigdb/collections.jsp>. Modules of disease are from <https://www.disgenet.org>. The details about these modules are also available at <https://github.com/TianyunC/cytokine-networks>.


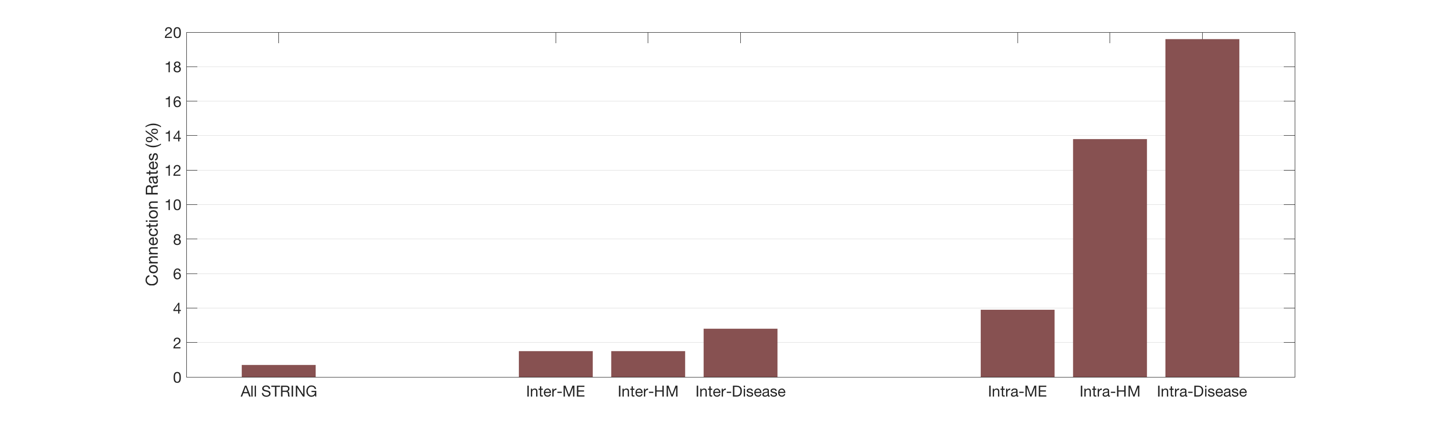


Fig B. The predicted Association Scores (Y-axis) of 8,521,944 edges correlate with their known STRING confidence scores. The grey area shows the standard deviation of predicted distances for a give confidence score. The correlation coefficient between the predicted Association Scores and the known STRING confidence scores is 0.554.

Fig C. The distribution of Association Scores (X-axis). We compared the distribution of predicted Association Scores (108,140,571 total values, shown in grey) against the 9,250,034 known confidence scores in STRING (shown in blue), of which 728,090 are high-confidence and 9,250,034 are low- or medium-confidence.

Fig D. Predicted Association Scores correlate with known confidence scores in STRING.

A total of 9,250,034 edges are known and assigned confidence scores by STRING (ranging from 100-1000). The differential box plots of Association Scores within each confidence bin indicate that Association Scores are positively correlated with STRING confidence scores.


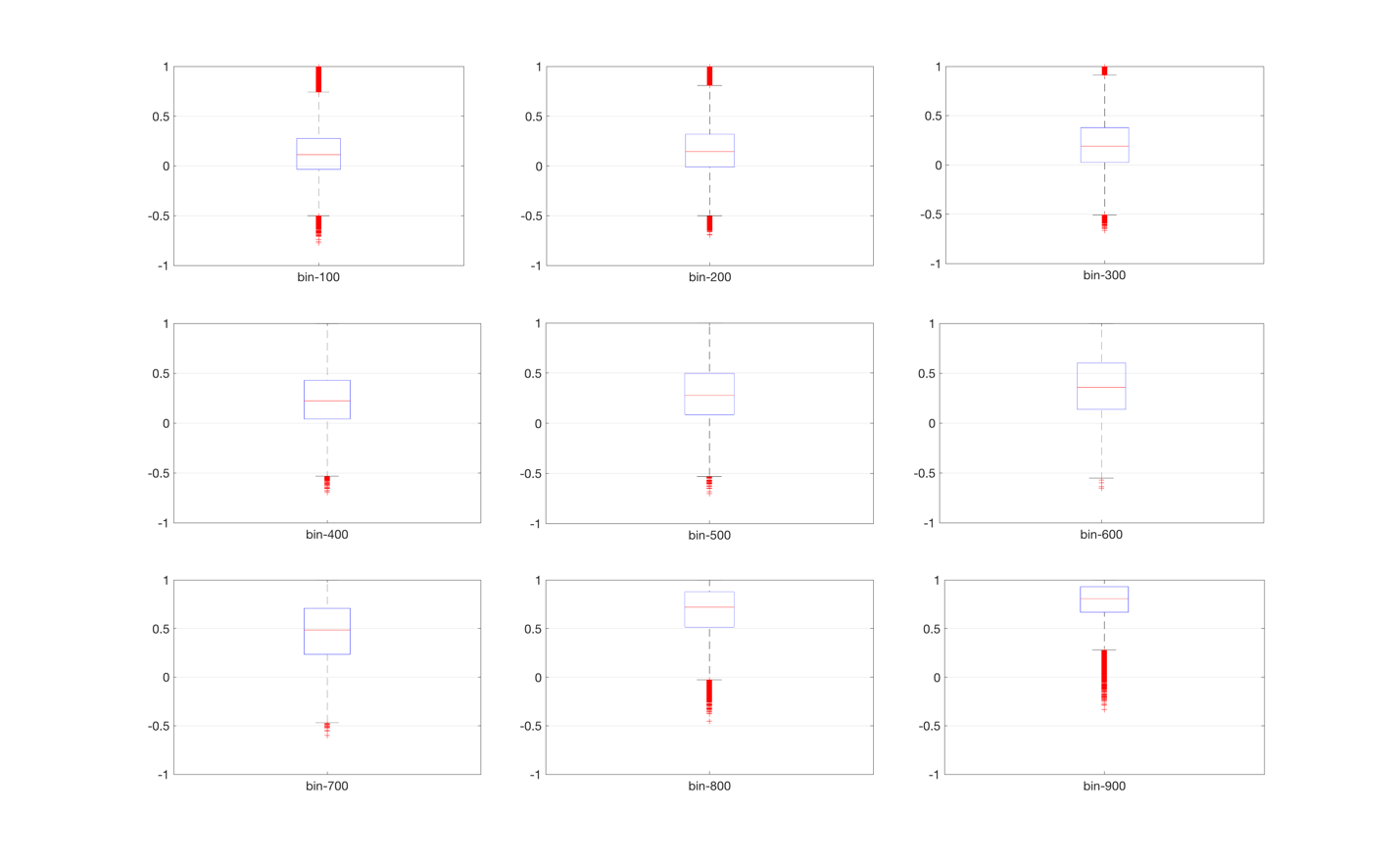


Fig E. The histogram of the number of genes associated with each of the 171 diseases.

Fig F The NAAS (X-axis) distribution within each bin defined by the number of genes associated with a disease. This was used to normalize the cytokine profile of individual diseases.

Fig G. The Number of genes associated with diseases is plotted against the P-value estimating the correlation between the predicted NAAS and the literature sampling frequency of cytokines. When the number of disease-associated genes increases, the accuracy of the predicted cytokine profiles decreases.

Fig H. The NAAS between aneurysm and each of the 79 cytokines are plot against the literature sampling frequency in aneurysm (data resource: ImmuneXpresso).

Fig I. Graph plots showing interactions between pathogenesis genes (green and purple squares) and inflammatory responses (essential genes in dots). The graph was plotted using a force-directed layout that uses attractive forces between adjacent nodes and repulsive forces between distant nodes. For immune disorder SLE, many pathogenesis genes (purple squares) are making interactions with core inflammation cytokines (orange) and chemokines (dark red) directly or through receptors (green squares). As for infectious disease TB, fewer pathogenesis genes (purple squares) are making interactions with inflammation cytokines (orange), and they are also farther away from chemokines (dark red). Attractive forces between pathogenesis and chemokine responses are observed in metabolic syndrome X, but not in aneurysm or acute leukemia.


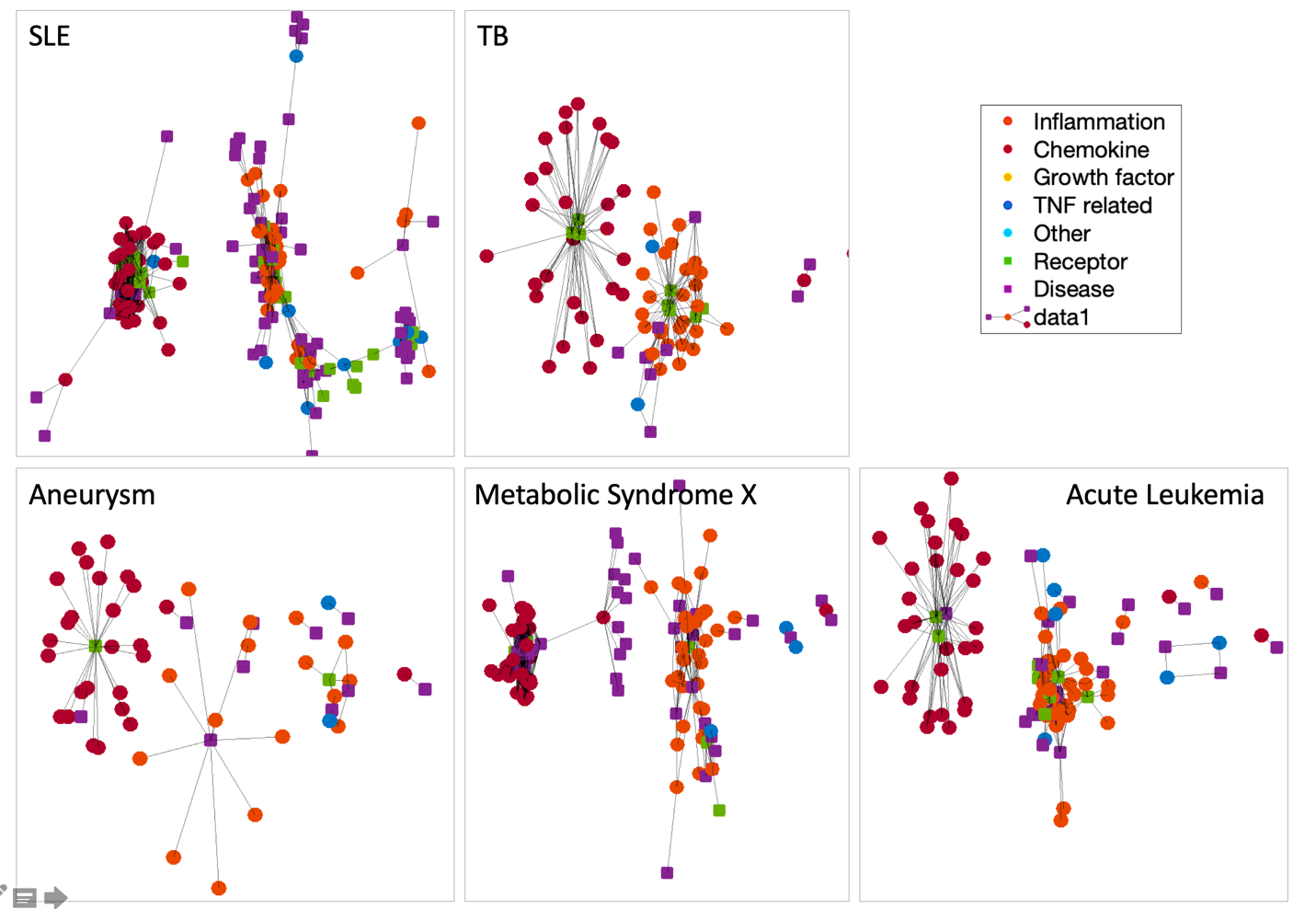


Fig J. Graph showing information flow from pathogenesis genes (green and purple squares) to inflammatory responses (essential genes in dots) by plotting the high-confidence interactions. The graph was plotted by placing genes into a set of layers, revealing their hierarchical structure.

Table A. Statistics of selected sets from STRING and three classes of functional modules. The fourth column ‘Reference” calculates the number of possible connections, while the fifth column shows the observed connection rate. When counting the connection observed in a gene set or between gene sets, we delete the genes observed in both sets.

| Subsets | # genes involved in edges with combined scores >0.8 | # Edges with combined scores >0.8 | Reference | Connection Rate |
| --- | --- | --- | --- | --- |
| STRING-human | 14707 | 728090 | 108140571 | 0.67 |
| 47 sets of ME | 5657 | 261520 | 15997996 | 1.63 |
| Between ME | 5378 | 209064 | 14023305 | 1.49 |
| Within ME | 3360 | 29958 | 770775 | 3.89 |
| 50 sets of HM | 3805 | 131420 | 7237110 | 1.82 |
| Between HM | 2013 | 30018 | 2126204 | 1.41 |
| Within HM | 1181 | 7264 | 56801 | 12.79 |

Table B. Gene signatures identified for the six groups of cytokines

| blue1 | RPS6, DOCK4, RPS9, RPS3A, RPS29, RPS28, RPS16, RPS21, RPS2, RPS3, RPS14, RPS23, RPS26, RPS18, RPS27L, RPS15, RPS11, MCTS1, RPS4X, RPS25, RPS19, RPS10, RPS24, RPS5, RPS17, RPS20, RPS7, FAU, RPS12, RPS13, RPS8, RPS15A, RPLP0, GSPT2, RPL8, RPL9, RPL32, RPL5, RPL12, RPL4, RPL13, RPL31, RPL18A, RPL7, RPL15, RPL30, RPL3, RPL6, RPL27, RPL11, RPL13A, RPL10A, RPL38, RPL26L1, RPL36, RPL35A, RPL10, RPL18, RPL28, RPL23, RPL37A, RPL17, RPL10L, RPL24, RPL19, RPL39, RPL26, RPL29, RPLP1, RPL3L, RPL7A, RPLP2, RPL39L, RPL22L1, RPL37, RSRC1, RPL22, RPL36A, RPL36AL, RPL21, RPL34, RPL14, RPL35, RPL27A, RPL23A, SRP54, C18orf32, RPL7L1, DIRC3, ETF1, SMG9, ABCF1, EIF3H, EIF4H, EIF5B, EIF3C, PELO, EIF3K, EIF3J, EIF3F, EIF3E, EIF3D, EIF1AX, EIF3M, EIF3L, EIF3CL, EEF1A2, SSR1, SSR3, SRPRB, TPT1, WDR31, SEC61A2, SEC11A, SEC61G, SRP72, ENSG00000215472, SRP14, SSR2, SRP68, SRP9, SPCS2, TRAM1, SERBP1, DENR, EIF5, EEF1D, ERI1, EIF3G, RPSAP58, SEC61B, SRP19, SRPR, SPCS3, SEC11C, SPCS1, NOP58, NOP56, BOP1, WDR12, PES1, RSL24D1, EIF6, HEATR1, PNO1, RRP9, DHX37, RPP30, RPP21, RPP14, RPP25, CIRH1A, BYSL, UTP3, NOL6, UTP14A, UTP11L, WDR75, DDX47, RRP36, RRP7A, EMG1, LTV1, RCL1, RIOK3, NOL11, UTP20, NOB1, FCF1, RIOK1, WBSCR22, UTP6, MPHOSPH10, WDR36, TBL3, UTP15, IMP4, PWP2, NOP14, KRR1, DDX52, NOC4L, BMS1, WDR43, UTP18, UTP14C, RIOK2, DDX49, DIEXF, WDR46, TSR1, WDR3, FBL, WDR18, EXOSC10, EXOSC3, EXOSC4, C1D, MPHOSPH6, NOL9, HBS1L, DIS3L2, DIS3, EXOSC1, EXOSC5, EXOSC8, EXOSC6, EXOSC7, EXOSC9, EXOSC2, PDCD11, MRTO4, IMP3, NSA2, RPP40, TTC37, ZBTB48, SUPV3L1, REXO4 |
| --- | --- |
| blue2 | KAT5, BTG1, RPA1, RPA2, RPA3, RFC5, RFC1, RFC2, RFC4, RFC3, POLE, POLE2, POLE3, ERCC1, ERCC4, POLE4, POLD3, POLD4, LIG1, MAD2L2, POLD1, DTL, CHRAC1, THUMPD1, WDR76, CHEK1, ATR, RAD51, RAD51C, BLM, TOP3A, BRCA2, EXO1, RAD51AP1, ATRIP, FANCD2, UBE2T, WDR48, USP1, FANCM, FANCE, FANCF, EME1, FANCG, POLN, FANCA, C19orf40, MUS81, SLX4, C17orf70, FANCB, FANCC, FANCL, EME2, C1orf86, DCLRE1B, DCLRE1A, FAN1, HELQ, WRN, RAD52, DNA2, XRCC3, XRCC2, RAD9B, RHNO1, SPIDR, RMI1, RAD51B, RAD17, RMI2, PALB2, TIMELESS, RAD1, HUS1, RAD9A, BRIP1, REV3L, RAD18, MSH2, MSH6, PIF1, MTMR12, |
| sig1 | APP, KNG1, PENK, C3, LPAR1, LPAR3, LPAR2, BDKRB1, CASR, MCHR2, NMUR1, LPAR5, PMCH, ANXA1, BDKRB2, NMU, NMUR2, NMS, GPR17, MCHR1, AGTR2, SAA1, C5, ADCY8, ADCY3, ADCY1, ADCY2, ADCY5, ADCY7, ADCY4, ADCY9, ADCY6, RLN3, GNAT3, SSTR3, GABBR2, HTR1B, GPR18, CCL28, HCAR3, NPW, TAS2R16, GRM8, RXFP3, NPY4R, NPY, SSTR2, CNR1, GRM6, GRM3, PTGDR2, APLN, SSTR4, ADORA3, P2RY13, TAS2R3, GPR31, TAS2R10, TAS2R7, CCR8, MTNR1B, OPRL1, S1PR5, RXFP4, TAS2R46, GPSM1, DRD3, GRM4, GPR183, HTR1A, CXCR5, TAS2R8, GPSM2, NPBWR1, HRH3, HTR5A, C5AR1, GRM7, GABBR1, SST, PNOC, TAS2R40, TAS1R1, TAS2R42, TAS1R3, CCR10, CCR9, SSTR1, TAS2R31, PYY, PDYN, TAS1R2, NPY1R, NPB, CCR4, TAS2R19, DRD4, APLNR, OPRK1, OXER1, OXGR1, CNR2, P2RY12, TAS2R4, NPBWR2, ADORA1, TAS2R1, TAS2R5, FPR3, TAS2R13, TAS2R60, INSL5, GPR55, NPY5R, HTR1E, SSTR5, HTR1F, TAS2R41, HTR1D, CORT, HCAR2, SUCNR1, TAS2R20, DRD2, GAL, TAS2R14, GPR37, PTGER3, CHRM4, TAS2R43, P2RY4, P2RY14, NPY2R, GALR1, HEBP1, GALR2, PCP2, GALR3, TAS2R39, TAS2R9, CXCR6, GRM2, TAS2R38, HRH4, TAS2R30, GPSM3, ACKR3, TAS2R50, CXCR2, OPRM1, HCAR1, CCR3, CXCR1, S1PR4, S1PR2, GPER1, S1PR3, ADRA2B, ADRA2C, CXCR3, CCL4L1, CX3CR1, MTNR1A, GPR37L1, OPRD1, PPY, CCR1, CCR2, ADRA2A, POMC, RGS13, RGS4, GPR61, PITPNM3, CXCR4, S1PR1, CCR5, CCR7, AGT, GNAI2, GNAI1, GNAI3, GPR78, RGS22, RGS5, PRKCD, EDN1, GNAZ, GNAO1, PRKCH, RGS1 |
| sig2 | STAT3, STAT6, STAT4, JAK1, TYK2, TBX21, STAT1, STAT5A, STAT5B, JAK3, IL13RA1, IL6R, IFNGR1, IFNGR2, SOCS3, SOCS1, PTPN1, PTPN2, KIT, IRS2, BCL6, JAK2, GH1, PRL, GH2, SOCS4, CSF2RA, CSF2RB, IL3RA, IL5RA, EPOR, SASH3, IKZF3, ENSG00000254469, IL2RG, IL2RB, IL2RA, NMI, STAT2, IFNA16, IFNA8, IFNA7, IFNA14, IFNA10, IFNA21, IFNA4, IFNA17, ENSG00000249624, IFNAR1, IFNE, IFNAR2, IFNW1, IL6ST, CNTF, LIFR, OSMR, IL4R, GHR, CRLF1, PRLR, CSH1, CTF1, PTPRE, MPL, IL15RA, IL10RB, IL22RA1, IL20RA, IL20RB, IL10RA, IL27RA, CNTFR, CRLF2, IL21R, IFNLR1, IL22RA2, IL11RA, CSH2, IL31RA, C19orf60, IL9R, CSF3R, IL12RB1, IL23R, IL12RB2, TSLP, IL13RA2, IRF9, USP41, IKZF1, KLRD1, KIR2DL3, KLRC1, KIR2DL1, KIR3DL1, LAG3, CD207, MRC2, ENSG00000255819, KLRK1, HCST, NCR1, NCR3, NCR2, KLRC4, IFNA5, SFTPC, SFTPB, SFTPD, SFTPA2, SFTA3, SFTPA1, FOXP4, PITPNA, FOXP2, GNLY, NKG7, PTPN5, FCER2, BATF, GLG1, GRAP, GAB4, FCRLA, FAM132B, TIE1, PTPRU, FLT3, STAP1, ALK, SH2B3, EML4, TFF2, DOK6, ANGPT2, ANGPT4, BTLA, VTCN1, DIRAS2, S100A13, LMO4, EOMES, PRF1, SP110, SPN, CR2, IKZF4, SOCS7, ICAM1, CD1B, CD274, CD28, CTLA4, SELL, CD19, CD34, CD5, MRC1, CD1E, CD1D, CD83, TNFRSF18, TNFSF18, ICOS, B7RP1, PTPRCAP, ICOSL, IL17RC, CLEC7A, FOXP1, CLEC1A, CD276, STAP2, HLX, RETNLB, IFNA13, TLR4, MYD88, IRAK4, IRAK1, TLR1, TLR2, IRAK2, IL1RAP, TLR9, TLR3, TIRAP, MALT1, IRAK3, TXNIP, MAP3K1, FASLG, MAP3K5, TNFRSF14, CD40, TNFRSF9, TLR7, TLR8, CNPY3, UNC93B1, TLR5, S100A8, IL1R2, IL1RL1, NRK, TMEM26, S100A9, LY86, FOXP3, CD27, CD69, SELE, IL1R1, IL18R1, IL18RAP, SIGIRR, IL37, IL18BP, LBP, KLF2, CCL3L3, GPR29, TNFRSF4, IDO1 |
| sig3 | UBA52, RPS27A, UBC, UBB, IFIT1B, DDI1, LYST, TMEM106B, TULP2, CTNNB1, YWHAE, YWHAG, YWHAB, PPP2CB, PPP2R1B, PPP2R5A, PPP2R5C, PPP2R5D, PPP2R5E, PPP2R5B, GSK3B, AXIN1, APC, AJUBA, CSNK1G2, CSNK1G1, FRAT2, FRAT1, GSK3A, APC2, MEA1, PMF1-BGLAP, COL4A3BP, NINJ1, EIF2AK4, C1orf111, DVL2, CFTR, CCDC88C, UBE2D1, ITCH, SMURF2, WWP1, SKP1, CUL1, SKP2, UBE2E1, CUL3, VHL, CUL2, FBXL7, ANAPC4, CDC23, ANAPC10, CDC26, ANAPC5, ANAPC2, CDC27, ANAPC7, ANAPC11, ANAPC1, FZR1, BTRC, UBE2C, CDC16, EPAS1, PSMB8, ANAPC15, ANAPC16, HIF3A, EGLN3, EGLN1, HIVEP3, PSMD1, PSMC4, PSMC5, PSMC2, PSMC1, CSNK1A1, AMER1, AXIN2, PSMA4, PSMC3, SHFM1, PSMD4, PSMD3, PSMD14, PSMB5, PSMA3, PSMA5, PSMA2, PSMB2, PSMD2, PSMA7, PSMD11, PSMD12, PSMA1, PSMB3, PSMD7, PSME3, PSMA6, PSMB4, PSMB9, PSMB11, PSMD13, PSMF1, PSMB1, PSMD5, PSME4, PSMD6, PSMB6, PSMD10, PSMD9, PSMC6, PSME1, PSMA8, PSMB10, PSMD8, PSMB7, PSME2, TP73, TNKS, TNKS2, MAPK6, PTGES3L, WTIP, LIMD1, SPOPL, PRICKLE1, ADRM1, RNF146, ZSWIM8, HECW1, UBD, PSMG2, CCDC74B, SPOP, OAZ2, POMP, KIAA2012, SMIM15, USP14, CCDC92, GNB2L1, HABP4, CASP4, EIF2AK1, GLI2, SUFU, GLI3, GLI1, IHH, DHH, CDON, PTCH1, BOC, GAS1, PTCH2, PAK2, NUMB, NUMBL, SHH, RUNX3, DISP1, AURKA, FBXO5, PTTG1, GTSE1, UCHL5, DZIP1, MYSM1, PARP6, KLHL12, ASNA1, SGTA, IGBP1, ENSA, CCNG2, PPP2R3C, SENP2, KLHDC3, QTRT1, QTRTD1, TXNL1, USP37, TBC1D20, USP6 |
| sig4 | APOB, TF, LAMB1, LAMC1, LAMB2, ADAM10, DNAJC3, FUCA2, P4HB, CTAGE6, HSP90B1, CDH2, QSOX1, CST3, HP, ORM2, APCS, SERPINA7, CKAP4, AHSG, GAS6, SERPINC1, IGFBP1, MSLN, MFI2, APOA5, PDIA6, MBTPS1, PNPLA2, CP, C4A, PRKCSH, TNC, CALU, IGFBP4, SPP2, CHRDL1, APOL1, PRSS23, EVA1A, FSTL3, IGFBP7, SCG3, MFGE8, AFP, STC2, DMP1, WFS1, VGF, FAM20C, APLP2, VWA1, AMTN, LTBP1, GOLM1, KTN1, MIA3, AMBN, HRC, TMEM132A, LGALS1, FSTL1, BPIFB2, ENAM, MEPE, MATN3, ITIH2, ANO8, SPARCL1, MXRA8, SERPIND1, SHISA5, SERPINA10, AMELX, SCG2, MGAT4A, FAM20A, CYR61, FBN1, IGFBP5, BMP15, NUCB1, PCSK9, NOTUM, CHGB, RCN1, PROC, IGFBP3, KLK4, APOA1, APOA2, GPC3, APOE, VCAN, MSR1, F2, CPB2, PROCR, SERPINA1, F5, F8, PAPPA2, TYRO3, VTI1B, THBS1, CSRP1, GP1BA, VTN, F10, SERPINA5, F9, F13B, GP5, GP9, GP1BB, F11, F12, KLKB1, SERPINE2, RIC8B, FN1, FGG, FGA, VWF, FGB, ACTN2, ACTN1, ACTN4, ALB, IGF2, KLK1, SERPINE1, A2M, CLU, SPARC, FERMT3, TMSB4X, PROS1, OLA1, F13A1, TEX264, SERPINF2, FAM49B, HRG, NHLRC2, SCCPDH, MMRN1, CFD, SRGN, MAGED2, GTPBP2, TOR4A, PCYOX1L, APOOL, LEFTY2, SERPING1, ISLR, DNAJC4, ALDOA, ORM1, PLG, FIGF, VEGFB, VEGFC, A1BG, TIMP1, REN, ACE, ENSG00000264813 |
| sig5 | GNB1, GNGT1, FPR2, GNG2, GNG13, GNG8, GNG11, GNG12, GNG7, GNB2, GNG4, GNGT2, GNB4, GNB3, GNB5, GNG10, GNG5, GNG3, CHRM2, GRK6, GNAL, C3AR1, PSAP, FPR1, PCSK1N, GPRC5C, TRPM5, RLN1, RTP4, OR13G1, ADORA2B, PTH, SLC5A1, KIAA1919, SLC15A1, RGS2, RGS21, GHRL, AGRP, PTAFR, ARR3, NLN, ARHGEF1, ARHGEF12, GNA12, CYTH4, ARHGEF5, AKAP13, VR1, AKAP1, GCG, NPS, P2RY11, GPR39, GCGR, GNAS, GNAQ, PLCB2, TBXA2R, GNA11, GNA14, EDNRA, GNA15, OXT, PLCB1, P2RY2, PTGER1, PLCB3, PLCB4, GNRH1, GNRH2, GPR132, AVPR1B, HCRT, CHRM5, EDNRB, XCR1, HCRTR1, QRFPR, CHRM3, AVPR1A, GHSR, F2RL3, OXTR, HTR2C, P2RY10, MLN, KISS1R, CCK, P2RY6, GPR143, TAC1, NPFFR2, KISS1, LPAR6, NMBR, GRPR, NMB, HTR2A, TACR2, LTB4R2, HCRTR2, FFAR4, NTSR1, PROK1, ADRA1B, PROKR2, UTS2R, GNRHR, GPR65, CYSLTR1, EDN3, F2RL1, GPRC6A, CYSLTR2, GRP, ADRA1D, QRFP, XCL2, MLNR, UTS2B, EDN2, GPR68, ADRA1A, CCKBR, LTB4R, PTGFR, CCKAR, FFAR2, BRS3, NPFF, NTS, FFAR3, TACR3, LPAR4, TRH, PROK2, UTS2, NTSR2, TRHR, GAST, HTR2B, NPFFR1, TAC3, ARHGEF25, P2RY1, HRH1, FFAR1, GPR4, OPN4, GRK5, GRM1, GRM5, CHRM1, F2R, F2RL2, ADRBK1, TRIO, KALRN, GNA13, KCNJ3, S100A11, CD68, ACE2, SPG7, PXYLP1, GALP, C9, C8B, C8A, C6, C8G, C7, PPIF, CHID1, CHI3L2, STAB1, ITIH4, CR1 |
| sig6 | TRAF2, BIRC3, BIRC2, NFKBIB, REL, RELB, MAP3K14, NFKBIA, NFKB2, TRAF3, TRAF6, TAB3, RIPK1, BCL10, TNFRSF1A, CARD11, RIPK2, NOD2, TICAM2, TICAM1, FADD, TRADD, TNFRSF25, FAS, RIPK3, IKBKG, IKBKB, CHUK, MAP2K4, MAP2K7, MAP3K3, MAP4K4, CRADD, TRAF1, TRAF5, TNFRSF10B, NSMAF, TNIK, BCL3, TLR6, PELI2, PELI1, PELI3, MAP3K1, FASLG, MAP3K5, TLR3, MALT1, TIRAP, TXNIP, NLRP3, SETD6, SETD4, NKIRAS2, RALGAPA1, GFOD2, GFOD1, DDX58, IFIH1, EIF2AK2, TBK1, MAVS, IKBKE, ZBP1, TANK, RNF135, AZI2, CMC1, NLRX1, BID, NOD1, MAP3K8, TNFAIP3, OTUD7B, USP2, CYLD, USP4, RNF31, SHARPIN, TAX1BP1, OTUB2, MADD, MLKL, PAWR, CARD10, CARD14, TNFRSF10A, TNFSF10, CFLAR, MAP4K2, TNFRSF17, PIDD1, TNFRSF10C, TNFRSF10D, TNIP1, IFI16, TBKBP1, ENSG00000026036, TNFRSF6B, TNFRSF13B, MAP3K7, USP21, TAB1, TAB2, XIAP, ERN1, CD14, LY96, TNIP2, PTPN11, GAB2, IRS2, PTPN1, PTPN2, KIT, TEK, ANGPT1, CSF1R, PLCG1, GRB2, SHC1, CRKL, PDGFRA, GAB1, IGF1R, PIK3CB, DIRAS1, PIK3CD, GDNF, IRS1, IRS4, BDNF, PRRT2, ERBB2, HBEGF, ERBB4, FLT1, SHC2, PTPN12, PGF, INSRR, FLT4, FGF13, CBLB, FCER1G, VAV2, VAV3, IGHV3-11, IGLL5, C6orf25, LCP2, ZAP70, GRAP2, FYB, SLA2, BLNK, PIK3AP1, DAPP1, SLA, IGHV4-38-2, NCAM1, ST8SIA4, LAT, UBASH3A, SYK, BTK, ITK, TRAT1, CD8A, CD8B, PAG1, BLK, PLCG2, CD79A, MS4A2, CD79B, FCGR2B, INPP5D, FCER1A, POU2AF1, FER, PDPN, INPPL1, BAG4, FES, PTPN13, BMX, DOK3, BCR, SOS2, NTF3, AXL, NTRK3, RIT1, SHC3, GRB7, NTF4, RIT2, RET, GFRA1, GFRA3, ARTN, PSPN, GFRA2, GFRA4, RAP1GAP, NRTN, DOK5, DOK1, SH2B2, DOK2, SHC4, GRB10, FLT3LG, KL, FGFR3, ZMYM2, MYO18A, FGF21, LRPPRC, TFF3, TNK2, TYROBP, TREM2, ENSG00000255641, TEC, SH3BP5, IBTK, CD22, KLRG1, CD72, CLEC1B, CD300LB, LAT2, TXK, IGLL1, VPREB3, TRPV6, SH2D2A, SLAMF1, CLEC6A, NIPSNAP1, UNC5C, UNC5D, PLEKHA2, RASGRP3, S100G, DOK4, LZTR1, CADPS2, LY6G6F, CD2, CD7, LGALS9, MME, CD48, MAP4K1, SH3BP2, CLEC4E |

Table C. The predicted cytokine profiles correlate with the known literature sampling frequency in ImmuneXpresso for the 171 well-studied diseases.

| Disease ID | #gene | p-value | CC | Disease name | Category | Category |
| --- | --- | --- | --- | --- | --- | --- |
| C1290886 | 41 | 8.78E-12 | 0.67525 | Chronic inflammatory disorder | C23 | "Pathological Conditions, Signs and Symptoms" |
| C0023290 | 74 | 9.39E-12 | 0.67454 | "Leishmaniasis, Visceral" | C01 | Infections |
| C0029118 | 29 | 1.39E-11 | 0.67044 | Opportunistic Infections | C01 | Infections |
| C1290884 | 174 | 3.88E-11 | 0.65944 | Inflammatory disorder | C23 | "Pathological Conditions, Signs and Symptoms" |
| C0275524 | 147 | 7.29E-11 | 0.65244 | Coinfection | C01 | Infections |
| C0026946 | 123 | 2.71E-10 | 0.63728 | Mycoses | C01 | Infections |
| C0035235 | 109 | 8.77E-10 | 0.62294 | Respiratory Syncytial Virus Infections | C01 | Infections |
| C0004623 | 234 | 1.85E-09 | 0.61346 | Bacterial Infections | C01 | Infections |
| C0011615 | 362 | 2.73E-09 | 0.60837 | Dermatitis, Atopic | C16;C17;C20 | "Congenital, Hereditary, and Neonatal Diseases and Abnormalities; Skin and Connective Tissue Diseases; Immune System Diseases" |
| C0243026 | 454 | 5.56E-09 | 0.59883 | Sepsis | C23;C01 | "Pathological Conditions, Signs and Symptoms; Infections" |
| C0009324 | 654 | 7.26E-09 | 0.59518 | Ulcerative Colitis | C06 | Digestive System Diseases |
| C0151317 | 5 | 7.59E-09 | 0.59457 | Chronic infection | C01;C20 | Infections; Immune System Diseases; |
| C0024530 | 396 | 9.74E-09 | 0.5911 | Malaria | C01 | Infections |
| C0021390 | 645 | 1.03E-08 | 0.59026 | Inflammatory Bowel Diseases | C06 | Digestive System Diseases |
| C0007570 | 220 | 1.25E-08 | 0.58761 | Celiac Disease | C06;C18 | Digestive System Diseases; Nutritional and Metabolic Diseases |
| C0036690 | 406 | 1.71E-08 | 0.58316 | Septicemia | C23;C01 | "Pathological Conditions, Signs and Symptoms; Infections" |
| C0019693 | 631 | 2.03E-08 | 0.58067 | HIV Infections | C01;C20 | Infections; Immune System Diseases |
| C0010346 | 622 | 2.30E-08 | 0.57884 | Crohn Disease | C06 | Digestive System Diseases |
| C0009319 | 516 | 2.31E-08 | 0.57878 | Colitis | C06 | Digestive System Diseases |
| C0155877 | 181 | 2.47E-08 | 0.57779 | Allergic asthma | C08;C20 | Respiratory Tract Diseases; Immune System Diseases |
| C0876973 | 70 | 3.38E-08 | 0.57319 | Infectious Lung Disorder | C01;C08 | Infections; Respiratory Tract Diseases |
| C2607914 | 131 | 3.38E-08 | 0.57316 | Allergic rhinitis (disorder) | C08;C20;C09 | Respiratory Tract Diseases; Immune System Diseases; Otorhinolaryngologic Diseases |
| C0242966 | 56 | 4.52E-08 | 0.56884 | Systemic Inflammatory Response Syndrome | C23 | "Pathological Conditions, Signs and Symptoms" |
| C0023281 | 56 | 5.36E-08 | 0.56624 | Leishmaniasis | C01;C17 | Infections; Skin and Connective Tissue Diseases |
| C0024131 | 252 | 8.27E-08 | 0.55958 | Lupus Vulgaris | C01;C17 | Infections; Skin and Connective Tissue Diseases |
| C0040100 | 180 | 9.23E-08 | 0.55789 | Thymoma | C04;C15 | Neoplasms; Hemic and Lymphatic Diseases |
| C0266929 | 119 | 1.06E-07 | 0.55569 | Chronic Periodontitis | C07 | Stomatognathic Diseases |
| C0025007 | 121 | 1.22E-07 | 0.55353 | Measles | C01 | Infections |
| C0026936 | 50 | 1.25E-07 | 0.55311 | Mycoplasma Infections | C01 | Infections |
| C0026918 | 88 | 1.27E-07 | 0.55281 | Mycobacterium Infections | C01 | Infections |
| C0036202 | 176 | 1.43E-07 | 0.55099 | Sarcoidosis | C15 | Hemic and Lymphatic Diseases |
| C0041327 | 135 | 1.51E-07 | 0.55013 | "Tuberculosis, Pulmonary" | C01;C08 | Infections; Respiratory Tract Diseases |
| C0042769 | 774 | 1.54E-07 | 0.54974 | Virus Diseases | C01 | Infections |
| C0014038 | 107 | 2.28E-07 | 0.54343 | Encephalitis | C10 | Nervous System Diseases |
| C0031099 | 157 | 2.36E-07 | 0.54288 | Periodontitis | C07 | Stomatognathic Diseases |
| C0031154 | 110 | 3.32E-07 | 0.53723 | Peritonitis | C06;C01 | Digestive System Diseases; Infections |
| C0041296 | 482 | 3.35E-07 | 0.53705 | Tuberculosis | C01 | Infections |
| C0011311 | 141 | 4.11E-07 | 0.53363 | Dengue Fever | C01 | Infections |
| C0023343 | 121 | 4.49E-07 | 0.5321 | Leprosy | C01 | Infections |
| C3714636 | 292 | 4.76E-07 | 0.53113 | Pneumonitis | C01;C08 | Infections; Respiratory Tract Diseases |
| C0023470 | 423 | 6.64E-07 | 0.52539 | Myeloid Leukemia | C04 | Neoplasms |
| C0019829 | 475 | 1.21E-06 | 0.51474 | Hodgkin Disease | C04;C20;C15 | Neoplasms; Immune System Diseases; Hemic and Lymphatic Diseases |
| C0003872 | 171 | 1.41E-06 | 0.51206 | "Arthritis, Psoriatic" | C17;C05 | Skin and Connective Tissue Diseases; Musculoskeletal Diseases |
| C0023418 | 1544 | 1.44E-06 | 0.51165 | leukemia | C04 | Neoplasms |
| C0021400 | 474 | 1.76E-06 | 0.50804 | Influenza | C01;C08 | Infections; Respiratory Tract Diseases |
| C0003864 | 518 | 1.94E-06 | 0.50621 | Arthritis | C05 | Musculoskeletal Diseases |
| C0033860 | 542 | 2.08E-06 | 0.50492 | Psoriasis | C17 | Skin and Connective Tissue Diseases |
| C0038013 | 194 | 2.21E-06 | 0.50382 | Ankylosing spondylitis | C05 | Musculoskeletal Diseases |
| C1264606 | 62 | 2.96E-06 | 0.49836 | Persistent infection | C23;C01 | "Pathological Conditions, Signs and Symptoms; Infections" |
| C0017152 | 141 | 3.05E-06 | 0.49774 | Gastritis | C06 | Digestive System Diseases |
| C0085669 | 425 | 3.61E-06 | 0.49454 | Acute leukemia | C23;C04 | "Pathological Conditions, Signs and Symptoms; Neoplasms" |
| C0042164 | 84 | 4.46E-06 | 0.4905 | Uveitis | C11 | Eye Diseases |
| C0023467 | 1378 | 4.49E-06 | 0.49037 | "Leukemia, Myelocytic, Acute" | C04 | Neoplasms |
| C0025289 | 53 | 5.05E-06 | 0.48805 | Meningitis | C10 | Nervous System Diseases |
| C0240066 | 58 | 5.08E-06 | 0.48795 | Iron deficiency | C18 | Nutritional and Metabolic Diseases |
| C0042384 | 97 | 5.78E-06 | 0.48543 | Vasculitis | C14 | Cardiovascular Diseases |
| C0011603 | 165 | 6.04E-06 | 0.48455 | Dermatitis | C17 | Skin and Connective Tissue Diseases |
| C1306759 | 145 | 6.14E-06 | 0.48423 | Eosinophilic disorder | C15 | Hemic and Lymphatic Diseases |
| C0006413 | 326 | 6.30E-06 | 0.48371 | Burkitt Lymphoma | C04;C01;C20;C15 | Neoplasms; Infections; Immune System Diseases; Hemic and Lymphatic Diseases |
| C0036117 | 34 | 6.69E-06 | 0.48254 | Salmonella infections | C01 | Infections |
| C0002940 | 136 | 6.82E-06 | 0.48214 | Aneurysm | C14 | Cardiovascular Diseases |
| C0026764 | 1056 | 9.31E-06 | 0.4759 | Multiple Myeloma | C04;C20;C15;C14 | Neoplasms; Immune System Diseases; Hemic and Lymphatic Diseases; Cardiovascular Diseases |
| C0079731 | 592 | 9.65E-06 | 0.47515 | B-Cell Lymphomas | C04;C20;C15 | Neoplasms; Immune System Diseases; Hemic and Lymphatic Diseases |
| C0024141 | 793 | 1.02E-05 | 0.474 | "Lupus Erythematosus, Systemic" | C17;C20 | Skin and Connective Tissue Diseases; Immune System Diseases |
| C0001175 | 140 | 1.13E-05 | 0.47198 | Acquired Immunodeficiency Syndrome | C01;C20 | Infections; Immune System Diseases |
| C0027059 | 99 | 1.19E-05 | 0.47085 | Myocarditis | C14 | Cardiovascular Diseases |
| C0008354 | 101 | 1.39E-05 | 0.46761 | Cholera | C01 | Infections |
| C0006118 | 541 | 1.46E-05 | 0.46665 | Brain Neoplasms | C04;C10 | Neoplasms; Nervous System Diseases |
| C0035243 | 47 | 1.48E-05 | 0.46628 | Respiratory Tract Infections | C01;C08 | Infections; Respiratory Tract Diseases |
| C0376545 | 413 | 1.79E-05 | 0.46237 | Hematologic Neoplasms | C04;C15 | Neoplasms; Hemic and Lymphatic Diseases |
| C0023434 | 870 | 2.30E-05 | 0.45696 | Chronic Lymphocytic Leukemia | C04;C20;C15 | Neoplasms; Immune System Diseases; Hemic and Lymphatic Diseases |
| C0524909 | 194 | 2.31E-05 | 0.45686 | "Hepatitis B, Chronic" | C06;C01 | Digestive System Diseases; Infections |
| C0024305 | 445 | 2.79E-05 | 0.45277 | "Lymphoma, Non-Hodgkin" | C04;C20;C15 | Neoplasms; Immune System Diseases; Hemic and Lymphatic Diseases |
| C0024299 | 1086 | 3.11E-05 | 0.45043 | Lymphoma | C04;C20;C15 | Neoplasms; Immune System Diseases; Hemic and Lymphatic Diseases |
| C0019163 | 656 | 3.16E-05 | 0.45008 | Hepatitis B | C06;C01 | Digestive System Diseases; Infections |
| C0023487 | 403 | 3.38E-05 | 0.44861 | Acute Promyelocytic Leukemia | C04 | Neoplasms |
| C0266999 | 112 | 3.76E-05 | 0.44624 | Vesicular Stomatitis | C01;C07;C22 | Infections; Stomatognathic Diseases; Animal Diseases |
| C0242497 | 1 | 3.98E-05 | 0.44497 | Intestinal schistosomiasis | C01 | Infections |
| C0026769 | 795 | 5.20E-05 | 0.43891 | Multiple Sclerosis | C20;C10 | Immune System Diseases; Nervous System Diseases |
| C0019348 | 422 | 6.26E-05 | 0.43466 | Herpes Simplex Infections | C01;C17 | Infections; Skin and Connective Tissue Diseases |
| C0039103 | 88 | 6.55E-05 | 0.43361 | Synovitis | C05 | Musculoskeletal Diseases |
| C0011854 | 705 | 7.05E-05 | 0.43191 | Diabetes Mellitus, Insulin-Dependent | C18;C20;C19 | Nutritional and Metabolic Diseases; Immune System Diseases; Endocrine System Diseases |
| C0149516 | 62 | 8.45E-05 | 0.42768 | Chronic sinusitis | C01;C08;C09 | Infections; Respiratory Tract Diseases; Otorhinolaryngologic Diseases |
| C0001339 | 88 | 8.74E-05 | 0.42687 | Acute pancreatitis | C06 | Digestive System Diseases |
| C0026948 | 102 | 9.01E-05 | 0.42615 | Mycosis Fungoides | C04;C20;C15 | Neoplasms; Immune System Diseases; Hemic and Lymphatic Diseases |
| C0023449 | 781 | 9.06E-05 | 0.42603 | Acute lymphocytic leukemia | C04;C20;C15 | Neoplasms; Immune System Diseases; Hemic and Lymphatic Diseases |
| C0018213 | 275 | 9.67E-05 | 0.42446 | Graves Disease | C11;C20;C19 | Eye Diseases; Immune System Diseases; Endocrine System Diseases |
| C0023473 | 764 | 0.00010036 | 0.42359 | "Myeloid Leukemia, Chronic" | C04;C15 | Neoplasms; Hemic and Lymphatic Diseases |
| C0036323 | 54 | 0.00010827 | 0.42177 | Schistosomiasis | C01 | Infections |
| C0019159 | 224 | 0.00011682 | 0.41994 | Hepatitis A | C06;C01 | Digestive System Diseases; Infections |
| C0023443 | 89 | 0.00014729 | 0.41429 | Hairy Cell Leukemia | C04;C20;C15 | Neoplasms; Immune System Diseases; Hemic and Lymphatic Diseases |
| C0024143 | 164 | 0.00017734 | 0.40968 | Lupus Nephritis | C13;C17;C12;C20 | Female Urogenital Diseases and Pregnancy Complications; Skin and Connective Tissue Diseases; Male Urogenital Diseases; Immune System Diseases |
| C3463824 | 575 | 0.00021196 | 0.4052 | MYELODYSPLASTIC SYNDROME | C15 | Hemic and Lymphatic Diseases |
| C0010823 | 232 | 0.00022847 | 0.4033 | Cytomegalovirus Infections | C01 | Infections |
| C0003873 | 1340 | 0.00027826 | 0.39823 | Rheumatoid Arthritis | C17;C05;C20 | Skin and Connective Tissue Diseases; Musculoskeletal Diseases; Immune System Diseases |
| C0001486 | 82 | 0.00028324 | 0.39778 | Adenovirus Infections | C01 | Infections |
| C0018799 | 228 | 0.00048032 | 0.38378 | Heart Diseases | C14 | Cardiovascular Diseases |
| C0024266 | 45 | 0.00052347 | 0.38145 | Lymphocytic Choriomeningitis | C01;C10 | Infections; Nervous System Diseases |
| C0023440 | 190 | 0.00053316 | 0.38094 | Acute Erythroblastic Leukemia | C04;C15 | Neoplasms; Hemic and Lymphatic Diseases |
| C3812396 | 8 | 0.00060645 | 0.37741 | Chronic idiopathic pulmonary fibrosis | C08 | Respiratory Tract Diseases |
| C0017658 | 145 | 0.00066619 | 0.3748 | Glomerulonephritis | C13;C12 | Female Urogenital Diseases and Pregnancy Complications; Male Urogenital Diseases |
| C0153690 | 293 | 0.00067075 | 0.37461 | Secondary malignant neoplasm of bone | C23;C04;C05 | "Pathological Conditions, Signs and Symptoms; Neoplasms; Musculoskeletal Diseases" |
| C0740457 | 341 | 0.00074817 | 0.37155 | Malignant neoplasm of kidney | C04;C13;C12 | Neoplasms; Female Urogenital Diseases and Pregnancy Complications; Male Urogenital Diseases |
| C0014175 | 511 | 0.00086264 | 0.36751 | Endometriosis | C13 | Female Urogenital Diseases and Pregnancy Complications |
| C0019196 | 629 | 0.00097739 | 0.36393 | Hepatitis C | C06;C01 | Digestive System Diseases; Infections |
| C2711227 | 424 | 0.0010794 | 0.36105 | Steatohepatitis | C06 | Digestive System Diseases |
| C0027697 | 108 | 0.0012002 | 0.35795 | Nephritis | C13;C12 | Female Urogenital Diseases and Pregnancy Complications; Male Urogenital Diseases |
| C0023493 | 426 | 0.0017368 | 0.34689 | Adult T-Cell Lymphoma/Leukemia | C04;C20;C15 | Neoplasms; Immune System Diseases; Hemic and Lymphatic Diseases |
| C0001815 | 147 | 0.0017583 | 0.34651 | Primary Myelofibrosis | C15 | Hemic and Lymphatic Diseases |
| C0036421 | 461 | 0.0017645 | 0.3464 | Systemic Scleroderma | C17 | Skin and Connective Tissue Diseases |
| C0155626 | 217 | 0.0018882 | 0.34433 | Acute myocardial infarction | C23;C14 | "Pathological Conditions, Signs and Symptoms; Cardiovascular Diseases" |
| C0677607 | 154 | 0.001936 | 0.34356 | Hashimoto Disease | C19 | Endocrine System Diseases |
| C0302592 | 907 | 0.0019578 | 0.34321 | Cervix carcinoma | C04;C13 | Neoplasms; Female Urogenital Diseases and Pregnancy Complications |
| C0024198 | 92 | 0.0019745 | 0.34295 | Lyme Disease | C01 | Infections |
| C0153676 | 580 | 0.0021391 | 0.34047 | Secondary malignant neoplasm of lung | C04;C08 | Neoplasms; Respiratory Tract Diseases |
| C0020443 | 198 | 0.0022024 | 0.33956 | Hypercholesterolemia | C18 | Nutritional and Metabolic Diseases |
| C0007222 | 657 | 0.0022104 | 0.33945 | Cardiovascular Diseases | C14 | Cardiovascular Diseases |
| C0948089 | 181 | 0.0029483 | 0.33032 | Acute Coronary Syndrome | C14 | Cardiovascular Diseases |
| C0524910 | 317 | 0.0033761 | 0.32593 | "Hepatitis C, Chronic" | C06;C01 | Digestive System Diseases; Infections |
| C0042373 | 340 | 0.0039602 | 0.32068 | Vascular Diseases | C14 | Cardiovascular Diseases |
| C0851162 | 1 | 0.0044806 | 0.31655 | Infections of musculoskeletal system | C23;C01;C05 | "Pathological Conditions, Signs and Symptoms; Infections; Musculoskeletal Diseases" |
| C0032463 | 170 | 0.0048689 | 0.31374 | Polycythemia Vera | C04;C15 | Neoplasms; Hemic and Lymphatic Diseases |
| C0030305 | 117 | 0.0052153 | 0.31139 | Pancreatitis | C06 | Digestive System Diseases |
| C0020456 | 416 | 0.0053691 | 0.3104 | Hyperglycemia | C18 | Nutritional and Metabolic Diseases |
| C0948008 | 351 | 0.0054805 | 0.30969 | Ischemic stroke | C10;C14 | Nervous System Diseases; Cardiovascular Diseases |
| C0036220 | 279 | 0.0065327 | 0.30358 | Kaposi Sarcoma | C04;C01 | Neoplasms; Infections |
| C0699791 | 1879 | 0.0083652 | 0.29476 | Stomach Carcinoma | C06;C04 | Digestive System Diseases; Neoplasms |
| C0038454 | 589 | 0.0084979 | 0.29419 | Cerebrovascular accident | C10;C14 | Nervous System Diseases; Cardiovascular Diseases |
| C0878544 | 330 | 0.010721 | 0.28563 | Cardiomyopathies | C14 | Cardiovascular Diseases |
| C0020459 | 238 | 0.010998 | 0.28468 | Hyperinsulinism | C18 | Nutritional and Metabolic Diseases |
| C3695127 | 2 | 0.012895 | 0.27865 | Astrocytoma of brain | C04;C10 | Neoplasms; Nervous System Diseases |
| C0585442 | 819 | 0.015445 | 0.27166 | Osteosarcoma of bone | C04 | Neoplasms |
| C3887641 | 12 | 0.016258 | 0.26964 | Recurrent hepatitis | C06 | Digestive System Diseases |
| C0020473 | 158 | 0.018798 | 0.26384 | Hyperlipidemia | C18 | Nutritional and Metabolic Diseases |
| C0524620 | 461 | 0.019872 | 0.2616 | Metabolic Syndrome X | C18 | Nutritional and Metabolic Diseases |
| C0006826 | 1061 | 0.02137 | 0.25863 | Malignant Neoplasms | C04 | Neoplasms |
| C0025202 | 1930 | 0.024963 | 0.25217 | melanoma | C04 | Neoplasms |
| C0149925 | 531 | 0.026365 | 0.24986 | Small cell carcinoma of lung | C04;C08 | Neoplasms; Respiratory Tract Diseases |
| C2939419 | 93 | 0.028499 | 0.24655 | Secondary Neoplasm | C23;C04 | "Pathological Conditions, Signs and Symptoms; Neoplasms" |
| C0035335 | 464 | 0.029609 | 0.2449 | Retinoblastoma | C04;C11 | Neoplasms; Eye Diseases |
| C0025517 | 367 | 0.033048 | 0.24012 | Metabolic Diseases | C18 | Nutritional and Metabolic Diseases |
| C0002170 | 108 | 0.033234 | 0.23987 | Alopecia | C23;C17 | "Pathological Conditions, Signs and Symptoms; Skin and Connective Tissue Diseases" |
| C0494165 | 420 | 0.033911 | 0.23899 | Secondary malignant neoplasm of liver | C06;C04 | Digestive System Diseases; Neoplasms |
| C0242339 | 174 | 0.034223 | 0.23858 | Dyslipidemias | C18 | Nutritional and Metabolic Diseases |
| C0027051 | 654 | 0.03449 | 0.23824 | Myocardial Infarction | C23;C14 | "Pathological Conditions, Signs and Symptoms; Cardiovascular Diseases" |
| C2239176 | 2810 | 0.048498 | 0.22273 | Liver carcinoma | C06;C04 | Digestive System Diseases; Neoplasms |
| C0699885 | 976 | 0.04937 | 0.22189 | Carcinoma of bladder | C04;C13;C12 | Neoplasms; Female Urogenital Diseases and Pregnancy Complications; Male Urogenital Diseases |
| C0017638 | 1748 | 0.054114 | 0.21755 | Glioma | C04 | Neoplasms |
| C0007137 | 1425 | 0.062044 | 0.21093 | Squamous cell carcinoma | C04 | Neoplasms |
| C0010054 | 645 | 0.078594 | 0.19908 | Coronary Arteriosclerosis | C14 | Cardiovascular Diseases |
| C0271650 | 261 | 0.085441 | 0.19476 | Impaired glucose tolerance | C18 | Nutritional and Metabolic Diseases |
| C1306459 | 847 | 0.096692 | 0.18821 | Primary malignant neoplasm | C04 | Neoplasms |
| C0011849 | 1243 | 0.10795 | 0.18224 | Diabetes Mellitus | C18;C19 | Nutritional and Metabolic Diseases; Endocrine System Diseases |
| C1140680 | 1668 | 0.1138 | 0.17933 | Malignant neoplasm of ovary | C04;C13;C19 | Neoplasms; Female Urogenital Diseases and Pregnancy Complications; Endocrine System Diseases |
| C0007131 | 1784 | 0.11705 | 0.17776 | Non-Small Cell Lung Carcinoma | C04;C08 | Neoplasms; Respiratory Tract Diseases |
| C0027819 | 1445 | 0.12619 | 0.17352 | Neuroblastoma | C04 | Neoplasms |
| C0699790 | 1771 | 0.12619 | 0.17352 | Colon Carcinoma | C06;C04 | Digestive System Diseases; Neoplasms |
| C0242379 | 1946 | 0.14719 | 0.1646 | Malignant neoplasm of lung | C04;C08 | Neoplasms; Respiratory Tract Diseases |
| C0043167 | 10 | 0.14894 | -0.16389 | Pertussis | C01;C08 | Infections; Respiratory Tract Diseases |
| C0018802 | 629 | 0.15792 | 0.1604 | Congestive heart failure | C14 | Cardiovascular Diseases |
| C0011860 | 1232 | 0.28004 | 0.12304 | Diabetes Mellitus, Non-Insulin-Dependent | C18;C19 | Nutritional and Metabolic Diseases; Endocrine System Diseases |
| C0235974 | 1552 | 0.3264 | 0.11185 | Pancreatic carcinoma | C06;C04;C19 | Digestive System Diseases; Neoplasms; Endocrine System Diseases |
| C0024121 | 598 | 0.35588 | 0.10526 | Lung Neoplasms | C04;C08 | Neoplasms; Respiratory Tract Diseases |
| C0001418 | 1298 | 0.35951 | 0.10447 | Adenocarcinoma | C04 | Neoplasms |
| C1458155 | 1506 | 0.41849 | 0.092302 | Mammary Neoplasms | C04;C17 | Neoplasms; Skin and Connective Tissue Diseases |
| C0686619 | 1039 | 0.43439 | 0.089195 | Secondary malignant neoplasm of lymph node | C23;C04 | "Pathological Conditions, Signs and Symptoms; Neoplasms" |
| C0678222 | 3876 | 0.5947 | 0.060772 | Breast Carcinoma | C04;C17 | Neoplasms; Skin and Connective Tissue Diseases |
| C0006142 | 3779 | 0.62734 | 0.055461 | Malignant neoplasm of breast | C04;C17 | Neoplasms; Skin and Connective Tissue Diseases |
| C0001430 | 828 | 0.79925 | -0.02907 | Adenoma | C04 | Neoplasms |
| C0020538 | 876 | 0.8853 | -0.016492 | Hypertensive disease | C14 | Cardiovascular Diseases |
| C0376358 | 2468 | 0.98317 | -0.0024114 | Malignant neoplasm of prostate | C04;C12 | Neoplasms; Male Urogenital Diseases |

Table D. The 171 diseases classified into three clusters based on their cytokine profiles

| Concept | Disease/Symptom | Class | Class Name |
| --- | --- | --- | --- |
| Cluster 1-2 (blue and cyan) | | | |
| C0014175 | Endometriosis | C13 | Female Urogenital Diseases and Pregnancy Complications |
| C0010054 | Coronary Arteriosclerosis | C14 | Cardiovascular Diseases |
| C0042373 | Vascular Diseases | C14 | Cardiovascular Diseases |
| C0948008 | Ischemic stroke | C10;C14 | Nervous System Diseases; Cardiovascular Diseases |
| C0027051 | Myocardial Infarction | C23;C14 | Pathological Conditions, Signs and Symptoms; Cardiovascular Diseases |
| C0038454 | Cerebrovascular accident | C10;C14 | Nervous System Diseases; Cardiovascular Diseases |
| C0007222 | Cardiovascular Diseases | C14 | Cardiovascular Diseases |
| C0018802 | Congestive heart failure | C14 | Cardiovascular Diseases |
| C0524620 | Metabolic Syndrome X | C18 | Nutritional and Metabolic Diseases |
| C0020456 | Hyperglycemia | C18 | Nutritional and Metabolic Diseases |
| C0020459 | Hyperinsulinism | C18 | Nutritional and Metabolic Diseases |
| C0020443 | Hypercholesterolemia | C18 | Nutritional and Metabolic Diseases |
| C0240066 | Iron deficiency | C18 | Nutritional and Metabolic Diseases |
| C0008354 | Cholera | C01 | Infections |
| C0153690 | Secondary malignant neoplasm of bone | C23;C04;C05 | Pathological Conditions, Signs and Symptoms; Neoplasms; Musculoskeletal Diseases |
| C0153676 | Secondary malignant neoplasm of lung | C04;C08 | Neoplasms; Respiratory Tract Diseases |
| C2939419 | Secondary Neoplasm | C23;C04 | Pathological Conditions, Signs and Symptoms; Neoplasms |
| C0494165 | Secondary malignant neoplasm of liver | C06;C04 | Digestive System Diseases; Neoplasms |
| C0023440 | Acute Erythroblastic Leukemia | C04;C15 | Neoplasms; Hemic and Lymphatic Diseases |
| C0271650 | Impaired glucose tolerance | C18 | Nutritional and Metabolic Diseases |
| C0020538 | Hypertensive disease | C14 | Cardiovascular Diseases |
| C0020473 | Hyperlipidemia | C18 | Nutritional and Metabolic Diseases |
| C0242339 | Dyslipidemias | C18 | Nutritional and Metabolic Diseases |
| C0018799 | Heart Diseases | C14 | Cardiovascular Diseases |
| C0035335 | Retinoblastoma | C04;C11 | Neoplasms; Eye Diseases |
| C0027819 | Neuroblastoma | C04 | Neoplasms |
| C1458155 | Mammary Neoplasms | C04;C17 | Neoplasms; Skin and Connective Tissue Diseases |
| C1140680 | Malignant neoplasm of ovary | C04;C13;C19 | Neoplasms; Female Urogenital Diseases and Pregnancy Complications; Endocrine System Diseases |
| C0017638 | Glioma | C04 | Neoplasms |
| C0007137 | Squamous cell carcinoma | C04 | Neoplasms |
| C0007131 | Non-Small Cell Lung Carcinoma | C04;C08 | Neoplasms; Respiratory Tract Diseases |
| C0699790 | Colon Carcinoma | C06;C04 | Digestive System Diseases; Neoplasms |
| C0699791 | Stomach Carcinoma | C06;C04 | Digestive System Diseases; Neoplasms |
| C0001418 | Adenocarcinoma | C04 | Neoplasms |
| C0686619 | Secondary malignant neoplasm of lymph node | C23;C04 | Pathological Conditions, Signs and Symptoms; Neoplasms |
| C0699885 | Carcinoma of bladder | C04;C13;C12 | Neoplasms; Female Urogenital Diseases and Pregnancy Complications; Male Urogenital Diseases |
| C0235974 | Pancreatic carcinoma | C06;C04;C19 | Digestive System Diseases; Neoplasms; Endocrine System Diseases |
| C0302592 | Cervix carcinoma | C04;C13 | Neoplasms; Female Urogenital Diseases and Pregnancy Complications |
| C0025202 | melanoma | C04 | Neoplasms |
| C1306459 | Primary malignant neoplasm | C04 | Neoplasms |
| C0006826 | Malignant Neoplasms | C04 | Neoplasms |
| C0740457 | Malignant neoplasm of kidney | C04;C13;C12 | Neoplasms; Female Urogenital Diseases and Pregnancy Complications; Male Urogenital Diseases |
| C0585442 | Osteosarcoma of bone | C04 | Neoplasms |
| C0149925 | Small cell carcinoma of lung | C04;C08 | Neoplasms; Respiratory Tract Diseases |
| C0024121 | Lung Neoplasms | C04;C08 | Neoplasms; Respiratory Tract Diseases |
| C2711227 | Steatohepatitis | C06 | Digestive System Diseases |
| C0011849 | Diabetes Mellitus | C18;C19 | Nutritional and Metabolic Diseases; Endocrine System Diseases |
| C0011860 | Diabetes Mellitus, Non-Insulin-Dependent | C18;C19 | Nutritional and Metabolic Diseases; Endocrine System Diseases |
| C0025517 | Metabolic Diseases | C18 | Nutritional and Metabolic Diseases |
| C0001430 | Adenoma | C04 | Neoplasms |
| C0878544 | Cardiomyopathies | C14 | Cardiovascular Diseases |
| C0242379 | Malignant neoplasm of lung | C04;C08 | Neoplasms; Respiratory Tract Diseases |
| C0376358 | Malignant neoplasm of prostate | C04;C12 | Neoplasms; Male Urogenital Diseases |
| C2239176 | Liver carcinoma | C06;C04 | Digestive System Diseases; Neoplasms |
| C0006142 | Malignant neoplasm of breast | C04;C17 | Neoplasms; Skin and Connective Tissue Diseases |
| C0678222 | Breast Carcinoma | C04;C17 | Neoplasms; Skin and Connective Tissue Diseases |
| Cluster 3 (green) | | | |
| C0036421 | Systemic Scleroderma | C17 | Skin and Connective Tissue Diseases |
| C0035243 | Respiratory Tract Infections | C01;C08 | Infections; Respiratory Tract Diseases |
| C0036690 | Septicemia | C23;C01 | Pathological Conditions, Signs and Symptoms; Infections |
| C0243026 | Sepsis | C23;C01 | Pathological Conditions, Signs and Symptoms; Infections |
| C0002940 | Aneurysm | C14 | Cardiovascular Diseases |
| C0948089 | Acute Coronary Syndrome | C14 | Cardiovascular Diseases |
| C0025289 | Meningitis | C10 | Nervous System Diseases |
| C0023281 | Leishmaniasis | C01;C17 | Infections; Skin and Connective Tissue Diseases |
| C0003872 | Arthritis, Psoriatic | C17;C05 | Skin and Connective Tissue Diseases; Musculoskeletal Diseases |
| C1290884 | Inflammatory disorder | C23 | Pathological Conditions, Signs and Symptoms |
| C0023343 | Leprosy | C01 | Infections |
| C0042164 | Uveitis | C11 | Eye Diseases |
| C0011603 | Dermatitis | C17 | Skin and Connective Tissue Diseases |
| C0876973 | Infectious Lung Disorder | C01;C08 | Infections; Respiratory Tract Diseases |
| C0017152 | Gastritis | C06 | Digestive System Diseases |
| C0017658 | Glomerulonephritis | C13;C12 | Female Urogenital Diseases and Pregnancy Complications; Male Urogenital Diseases |
| C0031099 | Periodontitis | C07 | Stomatognathic Diseases |
| C0266929 | Chronic Periodontitis | C07 | Stomatognathic Diseases |
| C0039103 | Synovitis | C05 | Musculoskeletal Diseases |
| C0031154 | Peritonitis | C06;C01 | Digestive System Diseases; Infections |
| C0027697 | Nephritis | C13;C12 | Female Urogenital Diseases and Pregnancy Complications; Male Urogenital Diseases |
| C0024143 | Lupus Nephritis | C13;C17;C12;C20 | Female Urogenital Diseases and Pregnancy Complications; Skin and Connective Tissue Diseases; Male Urogenital Diseases; Immune System Diseases |
| C1290886 | Chronic inflammatory disorder | C23 | Pathological Conditions, Signs and Symptoms |
| C0029118 | Opportunistic Infections | C01 | Infections |
| C0242966 | Systemic Inflammatory Response Syndrome | C23 | Pathological Conditions, Signs and Symptoms |
| C0001339 | Acute pancreatitis | C06 | Digestive System Diseases |
| C0026946 | Mycoses | C01 | Infections |
| C0011615 | Dermatitis, Atopic | C16;C17;C20 | Congenital, Hereditary, and Neonatal Diseases and Abnormalities; Skin and Connective Tissue Diseases; Immune System Diseases |
| C0033860 | Psoriasis | C17 | Skin and Connective Tissue Diseases |
| C0524910 | Hepatitis C, Chronic | C06;C01 | Digestive System Diseases; Infections |
| C0018213 | Graves Disease | C11;C20;C19 | Eye Diseases; Immune System Diseases; Endocrine System Diseases |
| C0041327 | Tuberculosis, Pulmonary | C01;C08 | Infections; Respiratory Tract Diseases |
| C0035235 | Respiratory Syncytial Virus Infections | C01 | Infections |
| C1306759 | Eosinophilic disorder | C15 | Hemic and Lymphatic Diseases |
| C0027059 | Myocarditis | C14 | Cardiovascular Diseases |
| C2607914 | Allergic rhinitis (disorder) | C08;C20;C09 | Respiratory Tract Diseases; Immune System Diseases; Otorhinolaryngologic Diseases |
| C0155877 | Allergic asthma | C08;C20 | Respiratory Tract Diseases; Immune System Diseases |
| C0042384 | Vasculitis | C14 | Cardiovascular Diseases |
| C3714636 | Pneumonitis | C01;C08 | Infections; Respiratory Tract Diseases |
| C0001175 | Acquired Immunodeficiency Syndrome | C01;C20 | Infections; Immune System Diseases |
| C0036220 | Kaposi Sarcoma | C04;C01 | Neoplasms; Infections |
| C0026948 | Mycosis Fungoides | C04;C20;C15 | Neoplasms; Immune System Diseases; Hemic and Lymphatic Diseases |
| C0014038 | Encephalitis | C10 | Nervous System Diseases |
| C0036202 | Sarcoidosis | C15 | Hemic and Lymphatic Diseases |
| C0149516 | Chronic sinusitis | C01;C08;C09 | Infections; Respiratory Tract Diseases; Otorhinolaryngologic Diseases |
| C3812396 | Chronic idiopathic pulmonary fibrosis | C08 | Respiratory Tract Diseases |
| C0242497 | Intestinal schistosomiasis | C01 | Infections |
| C3695127 | Astrocytoma of brain | C04;C10 | Neoplasms; Nervous System Diseases |
| C0155626 | Acute myocardial infarction | C23;C14 | Pathological Conditions, Signs and Symptoms; Cardiovascular Diseases |
| Cluster 4-5 (red and orange) | | | |
| C0001486 | Adenovirus Infections | C01 | Infections |
| C0006118 | Brain Neoplasms | C04;C10 | Neoplasms; Nervous System Diseases |
| C0023487 | Acute Promyelocytic Leukemia | C04 | Neoplasms |
| C0023473 | Myeloid Leukemia, Chronic | C04;C15 | Neoplasms; Hemic and Lymphatic Diseases |
| C0023467 | Leukemia, Myelocytic, Acute | C04 | Neoplasms |
| C0023418 | leukemia | C04 | Neoplasms |
| C0023449 | Acute lymphocytic leukemia | C04;C20;C15 | Neoplasms; Immune System Diseases; Hemic and Lymphatic Diseases |
| C0019163 | Hepatitis B | C06;C01 | Digestive System Diseases; Infections |
| C3463824 | MYELODYSPLASTIC SYNDROME | C15 | Hemic and Lymphatic Diseases |
| C0085669 | Acute leukemia | C23;C04 | Pathological Conditions, Signs and Symptoms; Neoplasms |
| C0023470 | Myeloid Leukemia | C04 | Neoplasms |
| C0376545 | Hematologic Neoplasms | C04;C15 | Neoplasms; Hemic and Lymphatic Diseases |
| C0006413 | Burkitt Lymphoma | C04;C01;C20;C15 | Neoplasms; Infections; Immune System Diseases; Hemic and Lymphatic Diseases |
| C0079731 | B-Cell Lymphomas | C04;C20;C15 | Neoplasms; Immune System Diseases; Hemic and Lymphatic Diseases |
| C0023434 | Chronic Lymphocytic Leukemia | C04;C20;C15 | Neoplasms; Immune System Diseases; Hemic and Lymphatic Diseases |
| C0024299 | Lymphoma | C04;C20;C15 | Neoplasms; Immune System Diseases; Hemic and Lymphatic Diseases |
| C0026764 | Multiple Myeloma | C04;C20;C15;C14 | Neoplasms; Immune System Diseases; Hemic and Lymphatic Diseases; Cardiovascular Diseases |
| C0024305 | Lymphoma, Non-Hodgkin | C04;C20;C15 | Neoplasms; Immune System Diseases; Hemic and Lymphatic Diseases |
| C0030305 | Pancreatitis | C06 | Digestive System Diseases |
| C0024530 | Malaria | C01 | Infections |
| C0275524 | Coinfection | C01 | Infections |
| C0010823 | Cytomegalovirus Infections | C01 | Infections |
| C0019693 | HIV Infections | C01;C20 | Infections; Immune System Diseases |
| C0009324 | Ulcerative Colitis | C06 | Digestive System Diseases |
| C0021390 | Inflammatory Bowel Diseases | C06 | Digestive System Diseases |
| C0010346 | Crohn Disease | C06 | Digestive System Diseases |
| C0026769 | Multiple Sclerosis | C20;C10 | Immune System Diseases; Nervous System Diseases |
| C0003864 | Arthritis | C05 | Musculoskeletal Diseases |
| C0036323 | Schistosomiasis | C01 | Infections |
| C0677607 | Hashimoto Disease | C19 | Endocrine System Diseases |
| C0026918 | Mycobacterium Infections | C01 | Infections |
| C0007570 | Celiac Disease | C06;C18 | Digestive System Diseases; Nutritional and Metabolic Diseases |
| C0023493 | Adult T-Cell Lymphoma/Leukemia | C04;C20;C15 | Neoplasms; Immune System Diseases; Hemic and Lymphatic Diseases |
| C0009319 | Colitis | C06 | Digestive System Diseases |
| C0019159 | Hepatitis A | C06;C01 | Digestive System Diseases; Infections |
| C0041296 | Tuberculosis | C01 | Infections |
| C0524909 | Hepatitis B, Chronic | C06;C01 | Digestive System Diseases; Infections |
| C0024198 | Lyme Disease | C01 | Infections |
| C0011311 | Dengue Fever | C01 | Infections |
| C0025007 | Measles | C01 | Infections |
| C0038013 | Ankylosing spondylitis | C05 | Musculoskeletal Diseases |
| C0024131 | Lupus Vulgaris | C01;C17 | Infections; Skin and Connective Tissue Diseases |
| C0019829 | Hodgkin Disease | C04;C20;C15 | Neoplasms; Immune System Diseases; Hemic and Lymphatic Diseases |
| C0023443 | Hairy Cell Leukemia | C04;C20;C15 | Neoplasms; Immune System Diseases; Hemic and Lymphatic Diseases |
| C0040100 | Thymoma | C04;C15 | Neoplasms; Hemic and Lymphatic Diseases |
| C0032463 | Polycythemia Vera | C04;C15 | Neoplasms; Hemic and Lymphatic Diseases |
| C0002170 | Alopecia | C23;C17 | Pathological Conditions, Signs and Symptoms; Skin and Connective Tissue Diseases |
| C1264606 | Persistent infection | C23;C01 | Pathological Conditions, Signs and Symptoms; Infections |
| C0019348 | Herpes Simplex Infections | C01;C17 | Infections; Skin and Connective Tissue Diseases |
| C0003873 | Rheumatoid Arthritis | C17;C05;C20 | Skin and Connective Tissue Diseases; Musculoskeletal Diseases; Immune System Diseases |
| C0011854 | Diabetes Mellitus, Insulin-Dependent | C18;C20;C19 | Nutritional and Metabolic Diseases; Immune System Diseases; Endocrine System Diseases |
| C0019196 | Hepatitis C | C06;C01 | Digestive System Diseases; Infections |
| C0042769 | Virus Diseases | C01 | Infections |
| C0024141 | Lupus Erythematosus, Systemic | C17;C20 | Skin and Connective Tissue Diseases; Immune System Diseases |
| C0021400 | Influenza | C01;C08 | Infections; Respiratory Tract Diseases |
| C0266999 | Vesicular Stomatitis | C01;C07;C22 | Infections; Stomatognathic Diseases; Animal Diseases |
| C0024266 | Lymphocytic Choriomeningitis | C01;C10 | Infections; Nervous System Diseases |
| C0036117 | Salmonella infections | C01 | Infections |
| C0023290 | Leishmaniasis, Visceral | C01 | Infections |
| C0004623 | Bacterial Infections | C01 | Infections |
| C0026936 | Mycoplasma Infections | C01 | Infections |
| C0151317 | Chronic infectious disease | C20 | Immune System Diseases |
| C3887641 | Recurrent hepatitis | C06 | Digestive System Diseases |
| C0001815 | Primary Myelofibrosis | C15 | Hemic and Lymphatic Diseases |
| Other (top and bottom, dark) | | | |
| C0851162 | Infections of musculoskeletal system | C23;C01;C05 | Pathological Conditions, Signs and Symptoms; Infections; Musculoskeletal Diseases |
| C0043167 | Pertussis | C01;C08 | Infections; Respiratory Tract Diseases |

Table E. Disease-associated genes in the well-connected modules formed by pathogenesis genes, receptors, and essential cytokines identified by spectrum partition on the interactions between disease-specific cytokine networks in the context of five immune disorders: rheumatoid arthritis (RA), psoriasis (PS), ulcerative colitis (UC), Crohn’s disease (CD) and systemic lupus erythematosus (SLE). Essential cytokines are marked with “E”; cytokine receptors are marked with “R”; other genes are marked with “D”.

| rheumatoid arthritis | | psoriasis | systemic lupus erythematosus | | ulcerative colitis | Crohn’s disease | |
| --- | --- | --- | --- | --- | --- | --- | --- |
| TNFRSF25 R  TRAF1 D  FASLG D  TNFRSF9 R  IL1A E  MAP3K5 D  TNFRSF1A R  MALT1 D  TLR4 D  TLR5 D  IL1RN R  IRAK1 D  S100A8 D  TLR1 D  NLRP3 D  TIRAP D  MYD88 D  TNF E  TLR2 D  TLR9 D  IL18RAP R  IL18R1 R  IL33 E  NFKBIA D  IL1R1 R  SIGIRR D  IL18 E  IL1B E  TNFSF4 E  TSLP D  FOXP3 D  IL23R R  STAT6 D  IL17A E  TBX21 D  IL13 E  IL10 E  IL10RA R  IL10RB R  IL4 E  IL12A E  IFNG E  IL21 E  IL24 E  IL19 E  IL20 E  IL22 E  IL26 E  IL11 E  IL12B E  IFNL1 E  IL27 E  IL5 E | IL7 E  STAT3 D  TYK2 D  IL4R R  IL9 E  OSMR D  JAK1 D  IL23A E  IL2 E  STAT5A D  STAT5B D  IFNGR1 R  STAT4 D  IL6R R  IL15 E  IFNGR2 R  IL2RA R  LIF E  CD70 E  IL2RG R  STAT1 D  IL31 E  IL2RB R  IL3 E  IFNL2 E  IL21R R  GH1 D  IFNLR1 R  IL27RA R  CSF2RA R  IL5RA R  IL9R R  IFNA6 E  IFNA2 E  PRL D  IKZF3 D  BCL6 D  SH2B3 D  SELE D  IFNA1 E  CTLA4 D  CR2 D  PTPN2 D  INPP5D D  IFI44 D  IFNL3 E  CLEC7A D  TNFSF9 E  MAP4K3 D  PDCD5 D  SPATA2 D  TNIP1 D  DDAH1 D | FASLG D  TNFRSF9 R  TNFSF15 E  TNFRSF17 R  TNFRSF10B R  TNFRSF13B R  TNFRSF6B R  TNFSF14 E  PSMD7 D  REL D  LTBR D  TNFRSF13C R  TNFSF13B E  TNFSF11 E | TRAF1 D  FASLG D  IL1A E  TNFRSF1A R  TLR4 D  IL1RN R  TLR5 D  IRAK1 D  S100A8 D  NLRP3 D  TIRAP D  MYD88 D  TNF E  TLR2 D  TLR9 D  IL18R1 R  IRAK4 D  NFKBIA D  IL1R1 R  IL33 E  IL1B E  SIGIRR D  IL18 E  TNFSF4 E  TNFRSF4 R  IL12RB1 R  FOXP3 D  IL23R R  IL17A E  TBX21 D  IL13 E  IL15 E  IL19 E  IL10RA R  IL10 E  IL2 E  IL4 E  IL22 E  IL23A E  IL12B E  IL12A E  IL10RB R  IL7 E  IL9 E  IL5 E  IL11 E  IFNG E  IL3 E  JAK1 D  STAT3 D | IL2RA R  IL4R R  TYK2 D  IFNL1 E  IL26 E  IL6R R  OSMR D  IL21 E  IL27 E  LIF E  IFNGR1 R  IFNGR2 R  STAT4 D  IL24 E  IL31 E  PRL D  IL21R R  IFNL2 E  STAT1 D  IL20 E  IFNLR1 R  IKZF3 D  CD70 E  SH2B3 D  IFNAR2 R  CR2 D  STAT2 D  SELE D  IFNA2 E  IFNA6 E  CTLA4 D  IFNA1 E  IFNK E  IRF9 D  IRF7 D  HLX D  IFNB1 E  IFIT1 D  IFI44 D  IRF1 D  IRF2 D  IRF5 D  IFNL3 E  CLEC7A D  TNFSF9 E  MAP4K3 D  SPATA2 D  TNIP1 D  ZC3H12A D | TNFRSF9 R  TNFSF15 E  TNFRSF17 R  CFLAR D  TNFRSF6B R  TNFSF14 E  BIRC2 D  BIRC3 D  NFKB2 D  USP14 D  REL D  PSMG1 D  EGLN3 D  TNFSF13B E  TNFRSF11A R  TNFSF11 E | TNFRSF9 R  IL1A E  MAP3K1 D  TNFRSF1A R  TNFSF4 E  TLR4 D  TLR5 D  IRAK1 D  TLR1 D  IL1RL1 R  IRAK3 D  NLRP3 D  IL1RN R  IL18RAP R  TLR2 D  TLR9 D  TNF E  IL33 E  NFKBIA D  IL18 E  IL1B E  FOXP3 D  TSLP D  STAT6 D  IL23R R  IL10RA R  IL17A E  IL10 E  IL13 E  IL10RB R  IL12A E  IL4 E  IL21 E  IFNG F  IL24 E  IL20 E  IL26 E  IL22 E | IL19 E  IL15 E  IL27 E  IL11 E  IL12RB2 R  IL7 E  IL5 E  IL12B E  IFNL1 E  IL23A E  IL4R R  IL9 E  TYK2 D  STAT3 D  GHR D  STAT5B D  STAT5A D  STAT4 D  LIF E  IFNGR2 R  IL2RA R  IL15RA R  IL31 E  IFNL2 F  IL2 E  IL3 E  CD70 E  GH1 D  JAK2 D  CSF2RA R  IFNA2 E  IFNA6 E  IFNA1 E  PTPN2 D  IFNL3 E  CTLA4 D  INPP5D D |

Table F: Frequency (#) in the five diseases.

| Gene | Type | # | Gene | Type | # | Gene | Type | # | Gene | Type | # |
| --- | --- | --- | --- | --- | --- | --- | --- | --- | --- | --- | --- |
| TNFRSF9 | R | 4 | IL18 | I | 3 | STAT5A | D | 2 | PSMG1 | D | 1 |
| TYK2 | D | 3 | IL17A | I | 3 | STAT1 | D | 2 | PSMD7 | D | 1 |
| TNFSF4 | T | 3 | IL15 | I | 3 | SPATA2 | D | 2 | PDCD5 | D | 1 |
| TNFRSF1A | R | 3 | IL13 | I | 3 | SIGIRR | D | 2 | NFKB2 | D | 1 |
| TNF | T | 3 | IL12B | I | 3 | SH2B3 | D | 2 | MAP3K5 | D | 1 |
| TLR9 | D | 3 | IL12A | I | 3 | SELE | D | 2 | MAP3K1 | D | 1 |
| TLR5 | D | 3 | IL11 | I | 3 | S100A8 | D | 2 | MALT1 | D | 1 |
| TLR4 | D | 3 | IL10RB | R | 3 | REL | D | 2 | LTBR | D | 1 |
| TLR2 | D | 3 | IL10RA | R | 3 | PTPN2 | D | 2 | JAK2 | D | 1 |
| STAT4 | D | 3 | IL10 | I | 3 | PRL | D | 2 | IRF9 | D | 1 |
| STAT3 | D | 3 | IFNL3 | F | 3 | OSMR | D | 2 | IRF7 | D | 1 |
| NLRP3 | D | 3 | IFNL2 | F | 3 | MYD88 | D | 2 | IRF5 | D | 1 |
| NFKBIA | D | 3 | IFNL1 | F | 3 | MAP4K3 | D | 2 | IRF2 | D | 1 |
| LIF | I | 3 | IFNGR2 | R | 3 | JAK1 | D | 2 | IRF1 | D | 1 |
| IRAK1 | D | 3 | IFNG | F | 3 | INPP5D | D | 2 | IRAK4 | D | 1 |
| IL9 | I | 3 | IFNA6 | F | 3 | IL6R | R | 2 | IRAK3 | D | 1 |
| IL7 | I | 3 | IFNA2 | F | 3 | IL21R | R | 2 | IL9R | R | 1 |
| IL5 | I | 3 | IFNA1 | F | 3 | IL1R1 | R | 2 | IL5RA | R | 1 |
| IL4R | R | 3 | FOXP3 | D | 3 | IL18RAP | R | 2 | IL2RG | R | 1 |
| IL4 | I | 3 | FASLG | D | 3 | IL18R1 | R | 2 | IL2RB | R | 1 |
| IL33 | I | 3 | CTLA4 | D | 3 | IKZF3 | D | 2 | IL27RA | R | 1 |
| IL31 | I | 3 | CD70 | T | 3 | IFNLR1 | R | 2 | IL1RL1 | R | 1 |
| IL3 | I | 3 | TSLP | D | 2 | IFNGR1 | R | 2 | IL15RA | R | 1 |
| IL2RA | R | 3 | TRAF1 | D | 2 | IFI44 | D | 2 | IL12RB2 | R | 1 |
| IL27 | I | 3 | TNIP1 | D | 2 | GH1 | D | 2 | IL12RB1 | R | 1 |
| IL26 | I | 3 | TNFSF9 | T | 2 | CSF2RA | R | 2 | IFNK | F | 1 |
| IL24 | I | 3 | TNFSF15 | T | 2 | CR2 | D | 2 | IFNB1 | F | 1 |
| IL23R | R | 3 | TNFSF14 | T | 2 | CLEC7A | D | 2 | IFNAR2 | R | 1 |
| IL23A | I | 3 | TNFSF13B | T | 2 | ZC3H12A | D | 1 | IFIT1 | D | 1 |
| IL22 | I | 3 | TNFSF11 | T | 2 | USP14 | D | 1 | HLX | D | 1 |
| IL21 | I | 3 | TNFRSF6B | R | 2 | TNFRSF4 | R | 1 | GHR | D | 1 |
| IL20 | I | 3 | TNFRSF17 | R | 2 | TNFRSF25 | R | 1 | EGLN3 | D | 1 |
| IL2 | I | 3 | TLR1 | D | 2 | TNFRSF13C | R | 1 | DDAH1 | D | 1 |
| IL1RN | R | 3 | TIRAP | D | 2 | TNFRSF13B | R | 1 | CFLAR | D | 1 |
| IL1B | I | 3 | TBX21 | D | 2 | TNFRSF11A | R | 1 | BIRC3 | D | 1 |
| IL1A | I | 3 | STAT6 | D | 2 | TNFRSF10B | R | 1 | BIRC2 | D | 1 |
| IL19 | I | 3 | STAT5B | D | 2 | STAT2 | D | 1 | BCL6 | D | 1 |
