# Supplementary material for: Construction of disease-specific cytokine profiles by associating disease genes with immune responses
Source: PLoS Comput Biol. 2022 Apr 11;18(4):e1009497. doi: 10.1371/journal.pcbi.1009497 (PMC9022887; doi:10.1371/journal.pcbi.1009497)
Supplement: Attachment — Submitted filename: Response2Reviewer.docx [file pcbi.1009497.s002.docx]

Reviewer's Responses to Questions

Reviewer #1: Here the authors ask, "how genes involved in pathogenesis which are often not associated with the immune system in an obvious way communicate with the immune system?". They further ask "What is the degree to which cytokine responses are shared across diseases or specific to each disease? And how does heterogeneity of cytokine responses mediate different pathogenesis and inflammatory processes?". These are important mechanistic questions. Their comprehensive analysis reveals subnetworks formed between disease-specific pathogenesis genes, hormones, receptors, and cytokines, leading to genes responsible for interactions between pathogenesis and inflammatory responses. In line with their observations, trials indicated a hierarchical framework of cytokines that defines groups for chronic inflammatory diseases rather differently from the homogenous molecular disease pattern previously assumed. The authors detail and annotate the genes that they identified and their functions. Altogether, this is a good, coherent paper that is well-suited to PLOS CB. Especially, I like it since it aims at elucidating mechanisms. It is always possible to raise technical, methodological and/or statistical questions. In this case I do not think that these are needed. The manuscript describes an in-depth, thoughtful, robust, and innovative approach and its output, and I hope that the results will indeed be useful toward drug discovery.

Thank you for your comments.

Reviewer #3: Manuscript PCOMPBIOL-D-21-01684
The article is framed in the potential relationship of inflammation on human disease and how to uncover this relationship. The authors present a method to obtain disease-specific cytokines and their associated disease-specific genes. The method is based on prediction of protein-protein interactions based on network embeddings and previous knowledge on inflammatory genes and disease genes. The manuscript would benefit on more clarity on the goals and description of the methodology. It has been hard to understand the rationale behind key aspects of the methodology and its implementation. Moreover, data and computational code is not available for evaluating the results and their reproducibility. Said that, from what I could understand, the proposed method is based on some assumptions that deserve a more careful thought. One is that there are disease-genes and disease-specific cytokines, and by mapping these gene sets to PPI networks it is possible to uncover relationships between inflammation and disease. This is fine with the exception that there are a lot of immune related genes and cytokines already associated to diseases. Thus, there is no such disjoint gene sets, and this is related to the complexity of biology and conflicts with our intention to classify processes in clear-cut boxes. The other caveat is the assumption of a complete graph for the protein interaction network. In my understanding biological networks are not complete graphs.

Thank you for your comments. Regarding your comments on the known cytokines in disease gene sets, we do have separate labels and used those to validate our predictions. Please see Table 2B. “The known DisGeNET genes of a given disease often contain cytokines already. The recall rate of these cytokines being recognized by the predicted subnetworks formed by high confidence associations ranges from 50% to 88%.” We do not aim to identify clear-cut boxes of immune responses. Instead, we evaluate the probability that a given set of disease genes is associated with an immune response. Regarding your concern about “a complete graph,” we evaluate the probability of association for all pairs of human genes, such that we can predict whether two genes are closely (high score) or remotely (low score) related.

1. A non-negligible number of disease genes are actually cytokines. How your method accounts for this overlap in gene sets? In this context, the separation of disease-gene and cytokine-gene sets seems rather artificial. Please elaborate on this.

We have separate labels for the cytokines identified as disease-related genes by DisGeNET and used those to validate our predictions. “The known DisGeNET genes of a given disease often contain cytokines already. The recall rate of these cytokines being recognized by the predicted subnetworks formed by high confidence associations ranges from 50% to 88%.” (Table 2B). We aim to identify genes that are closely related with cytokines from the pool of DisGeNET genes.

2. Intro, p. 4“Ideally, we would like to map known immune response genes more completely to human gene networks to better identify potential links to pathogenesis genes.” Do you mean inflammatory disease genes? There are many expressions like this in the article that are not clear enough to understand what the authors mean.

Thank you. We have modified the text in page 4 accordingly.

3. Why selecting STRING as a source of PPI interaction networks? There are other resources that are available and include the latest datasets for discovering PPIs, such as Intact. In addition, STRING includes different types of data and methods to derive the associations, the authors should specify which associations were included.

There are many ways to evaluate PPI and each has its own biases. STRING integrates observations from different resources and quantifies PPI by assigning scores based on the nature and quality of the supporting evidence. We feel that STRING is one of the most unbiased resources for PPI. We chose pairs with association confidence scores above 800 for training, and pairs below 800 for validation (page 15)

4. What is the rationale of the network sparsity analysis commented in the Intro? Why should we expect a complete graph in a biological network? This analysis should be better explained and justified.

We do not expect all genes to be associated as a complete graph. We aim to create a framework by which we can evaluate the probability of association between any pair of genes. The sparsity of current networks is biased by the availability of data, so we need predictions to fill those gaps.

5. Figure S1 refers to “modules”, but no details on how these modules are detected is provided. The same for the reference to “functional modules”. How immune response and disease modules are obtained, for instance? Please define the labels in Fig S1. Why the methodology for this analysis is not included in the methods section is not clear.

We have modified the legend of S1 accordingly. The estimation of network sparsity is simply made by calculating the ratio of the number of actual links to the total number of links present in a complete graph (explained in page 4). We feel that this is not the main contribution of the paper and did not include the method in the manuscript. Modules of ME are from ImmProt (Rieckmann, J. C. et al. Social network architecture of human immune cells unveiled by quantitative proteomics. Nat Immunol 18, 583-593, 2017). Modules of HM are from <http://www.gsea-msigdb.org/gsea/msigdb/collections.jsp>. Modules of disease are from <https://www.disgenet.org>. The details about modules are also available at <https://github.com/TianyunC/cytokine-networks>.

6. Does the Association Score consider experimental results for any given interaction?
Thank you. The Association Score is the cosine distance between the embedding vectors of two genes. We described the methods of Association Score in the Method section (page 15).

7. Figures S2-S4 refer to “network distance”, do you mean “association score”? It is quite difficult to follow the figures and the text is there is no consistency in the naming of variables.

Thank you. We have changed the text to Association Score.

8. Please explain which diseases were selected from DisGeNET (the 11,944 disease concepts) and the rationale for the selection.

Thank you. We have revised page 15. We have collected 11,944 disease concepts from DisGeNET ^21^, of which at least two genes and are associated with the disease.

9. Figure S5 is hard to interpret without labels in the axis.

Thank you. We have edited the figure accordingly.

10. The code and data are provided under this link <https://simtk.org/projects/cytokine>
But it is not possible to access to the data or code without being a member of the project. Therefore, the datasets and the code are not available to ensure reproducibility.

We have uploaded data files for public access. https://github.com/TianyunC/cytokine-networks

Reviewer #2: The manuscript entitled “Construction of disease-specific cytokine profiles by associating disease genes with immune responses” by Liu et al submitted to PLoS Computational Biology for publication (Manuscript Number: PCOMPBIOL-D-21-01684) presents an interesting attempt at discovering pathways of interaction between disease pathogenesis genes and immune-response driving cytokines and shed light on disease-specific mechanisms that mediate pathogenic immune-responses using an innovative network-based approach. However, there are a number of issues that need to be addressed before considering the manuscript for publication.

Thank you for your comments. We have made efforts to respond most of your comments by revising the text and adding more figures (in supplementary materials).

1. Page 3, lines 1-3: please provide references to support the claim made in the sentence.

Done.

2. Paragraph 5: please move the sentences describing the results presented in Supplementary Material Figure S1 either to the results section or to the method sections, where they can be used to justify the usage of the network embedding procedure rather than the STRING PPI-network itself.

Thank you for your comment. This is the background and motivation of our work, therefore we added it to the “INTRODUCTION” section.

3. Please represent the results presented in Figure 2A as a “STRING confidence score vs association score” density map scatterplot, so the reader can have a clearer idea of how those two metrics track each other. Also, please calculate a correlation coefficient and add it to the results.

We have included the scatter plot in Supplementary Material Figure S2, with a correlation coefficient calculated (0.554). However, we do not feel that the correlation coefficient for such a large set of data provides readers with a clear view of the Association Scores. Therefore, we keep the boxplot in the “RESULTS” and scatter plot in the “Supplementary Materials.” We modified the text in the “RESULTS,” such that readers can refer to the Supplementary Material if they need to.

4. Please perform a multiple-testing correction on the p-values obtained in the NAAS vs ImmuneXpresso literature co-occurrence frequency correlation analysis (Figure 4A) to decide which of the 171 disease show a statistically significant correlation.

Thank you. We have used the new p-value cutoffs derived from multiple testing correction. Please see revised Figure 4A.

5. Please represent the results presented in Figure 4B either as a Normalized Average Association Score vs literature co-occurrence frequency (in log scale?) scatterplot or, alternatively, as boxplot showing the distribution of NAAS for those disease-cytokine pairs with literature co-occurrence frequency above 0.005 and below 0.005 separately.

Thank you for your comments. We add a new figure (the scatter plot) as Supplementary Material Figure S7. We prefer to present the specific cytokines in the “RESULTS” section. We modified the text in “RESULTS” such that readers can refer to the Supplementary Material if they need to.

6. When presenting the results of the overlap between the high NAAS and high literature co-occurrence frequency, please add Specificity and overall Accuracy besides the recall.

Thank you for your comments. The literature co-occurrence does not serve as a perfect gold standard to evaluate our prediction, as many novel associations between cytokines and diseases are yet to be discovered. Therefore, calculating overall accuracy and recall could be misleading. We show the positive correlation between the predicted profiles and known associations to suggest that our predictions are reliable at some level.

7. Subsection “Defining the key modes of cytokine response”, paragraph 1:The authors state that “They fall into three distinct patterns in cytokine response”. According to what? The results of a hierarchical clustering? I know the readers can infer that from looking at Figure 5A but, by just reading the text, the assertion seems arbitrary. Please add detail.

We modified the text to “They fall into three clusters based on their interactions with cytokines (Figure 5A).”

8. The description of the results of Figure 5 in the main text and in the figure’s legend do not agree. Please revise so both pieces of text describe the results in the figure appropriately.

Thank you. We have revised and added labels for each cluster.

9. Subsection “Defining the key modes of cytokine response”, paragraph 2: It’s unclear what is meant by the first sentence. Please rephrase to make it clearer.

Thank you. Visually, it is clear that different families of cytokines interact with different groups of genes. This has been discussed in the previous section: “Essential cytokines can be classified into six clusters based on their interaction profiles.”

10. Please use ‘drive’ instead of decide in the third sentence of this paragraph.
    Thank you. We have revised accordingly.
11. In Figure 5A, it is not possible to identify the different groups of inflammatory cytokines (interleukins, interferons and TNFs). Please use color-coding on the cytokine labels to aid the identification of those subgroups of cytokines.

We worry that the figure will be confusing if we include more color themes. We rotated the figure for an enlarged view.

12. Please try and summarize the results presented in figure 5B more concisely. In general, immune disorder and infections, as expected, show higher values for all 4 cytokine-type components, with cardiovascular diseases presenting intermediate values, and metabolic diseases and, specially, neoplams, having the lowest average immune scores for all 4 components.

We have modified the text in page 8 accordingly.

13. Subsection “Inflammatory response subnetworks provide disease-specific insight”, paragraph 1: The fact that the subnetworks identified for each disease look different on visual inspection is a meaningful result. Please either remove that part of the sentences or elaborate in more detail into the differences between the disease subnetworks.

We have modified the text in page 8 accordingly.

14. Please explain the rationale for the assertion made in the last sentence of the paragraph. Would you expect to see more connections if, instead of cytokines, the interactors were metabolic or signaling genes? And, if so, what would you base your expectation on?

Thank you. We have clarified that this assertion is an observation made on our predicted associations.

15. Subsection “Inflammatory response subnetworks provide disease-specific insight”, paragraph 2: Please clarify if, for any given disease, only the cytokine receptors that are associated with the disease as per DisGeNET or, alternatively, all cytokine receptors that appear in your embedded network are included in these subnetwork.

We have added this in the text.

16. “Therefore, the ICD suggests the associations between…” please use some other term instead of “suggests”: “captures” or “represents” could do the job, for example.
    o It would be informative to assign p-values to the ICD values you calculate for each disease (Table 2A). For that, please, perform simulations, for each disease, by taking, randomly, as many non-cytokine genes in the network as the number of the DisGeNET genes for the disease that appear in your global network, calculate the ICD for the resulting subnetwork and, after repeating this procedure X times (at least 1000, preferably), calculate the percentile for your real ICD value to get a p-value. Repeating this procedure for each of the five diseases should assign a p-value for each ICD and aid its interpretation.

We believe the current calculation of ICD is sufficient to suggest an association density/frequency between pathogenesis and immune responses.

17. Subsection “Immune disorders pathogenesis genes connect to key inflammatory genes”, paragraph 1: In Figure 6, please explain in the legend that only NAAS values > 0.7 are shown. Also, please use 2-3 cytokines as examples to elaborate on and illustrate the fact that all 5 disease show similar patterns of association with inflammatory cytokines, but very varying levels with chemokines, TNFs and growth factors (Il18 and CCL17 look like they could be good candidates for that!).

We show the range between 0.7 to 1.0 for two reasons (1) we focus on associations with high scores (2) we need to space out the high-scored associations such that we can tell the difference. We added details accordingly.

18. Subsection “Immune disorders pathogenesis genes connect to key inflammatory genes”, paragraph 2:Page 10, line 1: there is probably a typo and “Box-I-1-4” should probably read “Box-I-1”. Please check and fix if necessary.

Thank you. We have corrected it.

19. From this point on, there are several instances where pre-inflammatory cytokines are mentioned. I assume this is a mistake and “pro-inflammatory” cytokines is the appropriate term. Please fix all instances if this is the case.

Thank you. We have corrected it.

20. Subsection “Highly connected graphs capture connections between immune disorder pathogenesis and inflammation”, paragraph 1:Is there a typo in the first sentence of is CD’s ICD really 0.71? Please check and amend if necessary.

Thank you. We have corrected it.

21. In supplementary table S5, it's impossible to infer from the gene-lists how many are shared across the diseases. Please add either venn diagrams showing the overlap between the gene-lists, or a list of unique geneIDs/symbols, indicating, for each gene, in how many diseases it appears and which are those diseases.

We have generated a new Table S5B that lists the frequency of each gene in the five diseases.

22. Discussion: Subsection “A complete map of associations between genes in large scale enables the identification of genome-wide features of cytokine interactions”, paragraph 1:It is difficult to understand the results presented in Supplementary Materials Table S1 without further explanation. For example, what do ME and HM stand for? How where those gene-sets obtained? Please add detail to aid understanding.

We have added details.

23. From the first four sentences of the paragraph, it doesn't follow that one needs to identify and explore novel associations between disease pathogenesis genes and the immune response. Please add another sentence in between to link the context provided at the beginning of the paragraph and the stated goal of the study.

We have addressed this goal in the “INTRODUCTION.” In addition, the 5^th^ sentence in the paragraph - “In this work, we aimed to identify novel associations between pathogenesis genes and immune responses” - serves this role.

24. Please explain why the fact that a network has "small world" properties makes it unsuitable for estimation of likelihood of association between genes via path-length calculations. For non-graph experts, this is not immediately obvious.

The STRING network itself is a highly-connected graph; the diameter of randomly generated graphs is approximately three or four steps. We have clarified that we cannot distinguish pairwise importance by the shortest path length, for there are too many pairs that share the same length. The embedding method allows us to distinguish pathways with higher resolution.

25. Subsection “Subnetworks between pathogenesis and inflammation suggest different mechanisms of immune response”: The results of Supplementary materials Figure S9 should be presented in the results section rather than here in the Discussion.

We reached the maximum number of figures allowed by the journal.

26. Subsection “Spectrum partition of subnetworks identifies key mediators of immune disorders”: “These key genes serve as candidates of therapeutic targets, as they are the main mediators to fuel inflammation”: This is a very strong assertion that, I feel, is not justified by the results. It is true that the subnetworks have allowed the identification of genes involved in pathogenesis that might have a notorious role in driving the immune responses in those diseases, but more work is needed to confirm that this is, indeed, the case, and that they could be good candidates for therapeutic intervention. Please rephrase to tone down.

We have modified the manuscript accordingly.

27. “The human trials targeting different cytokines suggest the existence of a hierarchical framework of cytokines that defines groups for chronic inflammatory diseases rather differently from the homogenous molecular disease pattern previously assumed”: I find this sentence very difficult to understand. Please rephrase to increase clarity.

We have modified as below.

The human trials targeting different cytokines showed differential efficacy of cytokine inhibition in chronic inflammatory diseases. Most of the chronic inflammatory diseases share clinical responsiveness to TNF-a inhibition but differ in their responsiveness to inhibition of cytokines, such as IL6, IL1, IL17 and IL23. This suggests the existence of a hierarchical framework of cytokines that defines groups for chronic inflammatory diseases rather differently from the homogenous molecular disease pattern previously assumed. We have clarified this in the discussion.

28. “These observations validate our predicted key mediators for pathogenesis and inflammation”: I think “validate” is a clear overstatement here. Please tone down to “support” or to another similar term.

We have modified the manuscript accordingly.

29. Please add a paragraph discussing the limitations of the study and another one discussing potential future steps regarding how to overcome those limitations and/or strategies for validation of the therapeutic candidate genes identified.

We have added text accordingly.

30. Methods: Please provide details on the experiment performed to find the optimal hyper-parameters for the embedding algorithm and how the final parameters were chosen.

We have added method notes in Supplementary Material.

31. Did you calculate the cosine distance or the cosine similarity? I mean, if the resulting Association Scores are positively associated with the edge-confidences in STRING, I suppose that cosine similarities were calculated, rather than distances (1-cos_similarity). Otherwise, the paper doesn't make any sense. Please clarify.

One of the Distance Metrics (in MatLab and other systems too) is “cosine”. So when we say “cosine distance”, it means “cosine similarity”, even though “cosine distance” is sometimes defined as 1-“cosine similarity”. We have modified the text anyway.

32. As far as I can tell, there are 140 cytokines in ImmuneXpresso. Did only 126 of those map onto the 14707 human gene network? Please clarify.

Some of the cytokines do not have high confidence connections in STRING and therefore cannot be embedded.

33. Please add explanations on why it is necessary to perform the embedding of the STRING network onto 64-dimensional space rather than using the STRING network itself to calculate potential disease-gene – cytokine associations.

As we have discussed in the section “Discussion”: The STRING network itself is a highly connected graph that obeys “small world” statistics and thus path length calculations are not useful for estimating likelihood of association^21^. We cannot distinguish pairwise importance by the shortest path length, for there are too many pairs that share the same length. The embedding space provides more information by capturing the topological structures of STRING, enabling a complete map of pairwise associations.

34. Why was necessary to normalize the average association scores for each cytokine and disease for gene-set size? Did you observe an association between the number of genes associated to a disease and the average Association Scores between the genes and the cytokines? If so, please provide some result showing this association.

This is shown in Supplementary Material Figure S5-B, which shows that the scores have different distribution when diseases are grouped by their associated genes. That is why we used the distribution of each group for normalizations of cytokine profile of individual diseases (page 16, secondary paragraph).

35. It is not clear to me how the cytokine profiles are normalized by the associated-gene-count-bin each disease falls into. For starters, in each bin, is the calculation of the p-value done using the distribution of the association score across all cytokines or for each cytokine separately? Also, how is the normalized average Association Score calculated, then? Is it the -log10pvalue? Or the percentile? Please add details to clarify.

It is just as stated in the text: [number of diseases of which average cosine similarities <given distance] / [total number of diseases in the bin].

36. For the validation of the NAAS scores, did you calculate the Spearman correlation between the NAAS profile across 79 cytokines for a disease and the corresponding profile of the literature sampling frequency for the disease? Please add clarification. Also, please explain the rationale behind using ImmuneXpresso's literature sampling frequency profile to validate the Association Score and an explanation of what the “literature sampling frequencies” represent. Are these frequencies of co-occurrence of a disease and a cytokine across the literature?

We have modified to clarify. As to the “literature sampling frequencies” provided by ImmuneXpresso, it was calculated by their NLP algorithms. We don’t feel the need to write details of methods that were published by other researchers, as the reference to the original paper would provide all the details.

37. How was the filtering done to reach the list of 110 cytokine receptors from GeneCards? Please provide details.

In GeneCards advanced search, we used search “receptor” in “aliases and description” and “immune response” in “functions” to retrieve 428 receptors. We then manually refined the list to 110 cytokine receptors.

38. “and is the cosine distance of two sets of genes in embedding space”. Two comment here: Again, please clarify if you calculated cosine distances or cosine similarities, as the analysis wouldn’t make sense as it stands if using the former.

We have modified accordingly.

39. I would expect the dpi to represent the cosine distance (or similarity) in embedding space between each pair of pathogenesis genes and cytokines and not the overall cosine distance (or similarity) between the two sets of genes, right? Please clarify.

No. In the figure legend of Figure 7, it says that “The graph was plotted in force-directed layout that uses attractive forces between adjacent nodes and repulsive forces between distant nodes. The distances between two vertices are roughly proportional to the length of the shortest path between them.”. We added more details in the method section (page 17). “We graphed the subnetwork formed by high confidence connections (Association Score > 0.8) between disease genes and cytokines in a force-directed layout that uses attractive forces between adjacent nodes and repulsive forces between distant nodes. The distances in the graph between two vertices are roughly proportional to the length of the shortest path between them within the subnetwork.”

40. If the object of the spectrum partition was to identify the pathogenesis genes showing a high connectivity with cytokines in each disease-specific subnetwork, I would expect one would have to remove all the interactions among pathogenesis genes, too, and not only the interactions among cytokines as, otherwise, the Fiedler vectors will reflect not only pathogenic gene – cytokine connectivity but, also, pathogenic gene – pathogenic gene connectivity. However, the text only makes reference to interactions between cytokines being removed. Please clarify this point.

Yes. Those have been removed. We have modified the text to clarify.

41. Figures: In Figure 1A, please remove the arrow from the “14,707 genes” box to the “9,250,034 pairs in STRING” box as it suggests that the STRING interactions were identified based on the initial selection of those genes when, according to the explanation in the methods section, that is not the case.

Modified accordingly.

42. Overall comments: Throughout the text inflammation/inflammatory and immune/immunological seem to be used interchangeably. My understanding is that the scope of the study is the exploration of the broader immune response rather than of the particular inflammatory aspect of immune responses. If that’s the case, please change all instances where inflammation/inflammatory is used to refer to the broader immune response. Otherwise, please clarify in the text that the study’s focus is inflammation only.

Cytokines are functional molecules of inflammation, and so we make generic reference to inflammation or inflammatory response in the paper. However, we understand the reviewer’s point, and so we reviewed the whole manuscript and made changes where “immune response” should be used for general discussion. Thank you.

43. There are a few instances in the text where the study is framed as identifying disease-specific cytokine response profiles. I find this misleading. No levels of cytokines or of their downstream effectors were used in the study. Please rephrase those instances to more accurately reflect the fact that the cytokine profiles represent network-interaction-based potential cytokine involvement rather than cytokine response.

We are predicting the probability of association between cytokines and a disease. We find different probabilities of association for different diseases, leading to a kind of cytokine qualitative “fingerprint.” This is the sense in which we mean disease-specific profile.

44. There are quite a few grammatical errors and some of the sentences are not worded in an easily understandable manner. Please get the manuscript proofread by a native English speaker to fix grammatical and punctuation mistakes and make some sentences clearer.

We have carefully reviewed the manuscript and tried to correct any grammatical and punctuation mistakes.

45. In this work, the concept of seeking to understand how genes involved in pathogenesis which are often not associated with the immune system in an obvious way communicate with the immune system is an endeavor of interest to the research community. Overall, the writing can be further refined for more concise and specific articulation and flow of concepts. It is a useful endeavor to establish a threshold of high confidence interactions. Even in the case where the interaction can occur: ligand/receptor, it is not an indication that in a given disease tissue it does occur unless covariance and level of expression signifies there is a high probability or confidence level of this occurring in which case rna and protein expression would have to be used as supporting data.

Our goal is the prediction associations between genes. We fear it would be misleading if we try to infer “binding” or “co-expression”. Validating such prediction based on data from a certain type of experimental resource is not right.

46. Another question to ask is how does the hierarchy of cytokine response differ or is conserved across diseases and can you characterize the various function all gene sets that are associated with specific cytokine responses. This was not done. It remains unclear whether inflammation score is differentiating/qualifying the type of inflammatory response or whether it is quantifying the magnitude of immune response.

Because of the limitations of data and methods, we cannot derive such information. We added this in the discussion.

47. Unclear what the rationale is for merging all diseases in the analysis between protein protein interaction and disease genes and then separating the diseases for inflammation score. The former may be dependent on the disease with some genes interacting in some diseases and not others whereas the magnitude of inflammation score may converge across diseases since it tracks with severity of inflammation. Gene interactions may also depend on tissue context which is associated with disease type. It is these context dependent interactions which may be the drivers of cytokine responses, with some collections of cytokines triggering dependencies on other cytokines so it maybe overly simplistic to start with one universal disease gene PPI framework. In this way, the ingoing assumption is flawed.

We absolutely agree that there are tissue-specific effects that are lost in using a unified PPI network. This is a first effort to make these inferences, and so we have used the best resource available. As tissue-specific networks are made available, our future work should focus on using tissue-specific PPI networks to refine our predictions, but in this first pass, we combined all interactions. We agree that this strategy could lead to some false positive predictions. We have modified the text to indicate this in the discussion.

48. The ‘known’ disease cytokine associations derived from NLP are questionable.
    • Provide a build in of an in silico negative control.
    • It would be interesting if this model could parse the relationships between what disease genes are causal to driving a cytokines, how the inter relationship of different cytokines are altered in different disease settings and then what is the disease gene effect of cytokine stimulation.

Unfortunately, we did not find better resources about cytokines other than the NLP-based ImmuneXpresso.

49. • Some of the preliminary conclusions have not been vetted properly to identify biological meaning. For example ” Chemokine-CLU is associated with a set of genes that function in G-protein signaling pathway and the response to endogenous and environmental insults ". This is the case because chemokine receptors are GPCRs.
    • “Note that of the twenty neoplasms in cluster-3, nineteen are hematic and lymphatic diseases (C15/C04), suggesting that these neoplasms have distinct cytokine distributions from other neoplasms. This is expected given hematopoietic and lymphatic are immune organs as compared to neoplasms of other tissues.
    • “We also found that genes responsible for Ubiquitin proteasome pathways interact with TNFs, not other cytokines” already known in part and not entirely true.
    • Main conclusions are either too vague or already known or recite existing open questions which have not been sufficiently investigated and analyzed in this model.
    • Neoplasms (systemic lupus erythematosus (SLE), TB, aneurysm, metabolic syndrome X, and acute leukemia). These examples do not fit the definition of neoplasm.
    • While the collection of methods are interesting, the novel conclusions are limited, i.e it is already known TLR activation is linked to cytokine/TNF response.
    • Since RA, SLE and IBD are inflammatory diseases it is expected that they would have a higher ICD score than the other disease genes. The final conclusions between these diseases is vague and not analyzed with sufficient depth.

Thank you for your comments. Our goal is to provide new biology using limited data resource about cytokines. Some of the findings may be “re-discovery” (GPCRs), which we consider positive controls that build confidence in our method. We chose to present our findings this way because further validation using PubMed may be circular or lead to biased conclusions. We have observed that if we Google any predicted pairs of a cytokine and a disease, we would be very likely to see some published evidence for “validation”. Regarding your question about neoplasm, we did not define SLE, TB, aneurysm as neoplasms. Thank you for your remarkably detailed and thoughtful comments.
