# Supplementary material for: Construction of disease-specific cytokine profiles by associating disease genes with immune responses
Source: PLoS Comput Biol. 2022 Apr 11;18(4):e1009497. doi: 10.1371/journal.pcbi.1009497 (PMC9022887; doi:10.1371/journal.pcbi.1009497)
Supplement: Attachment — Submitted filename: Revision2.docx [file pcbi.1009497.s003.docx]

Dear Editor,

Thank you.

We have processed all our figures using PACE.

Below is our response to reviewer #2.

Reviewer #2: Please address the remaining issues in your analysis:

1. The pathogenesis genes which you cite as interacting with cytokines (immune genes) are in fact almost all immune genes because there are inflammatory diseases (Table 3)

The definition of “immune gene” has not been well defined. The fact that most of these genes are related to immune responses suggests the reliability of predictions. We modified the table legend accordingly.

2. CVD may not be an infectious or autoimmune disease, but it is widely considered a chronic inflammatory disease. Aneurysm or leukemia may affect blood or vessel lining cells but they are not considered as immune mediated per se.

We agree. Our predictions quantify their immune scores, aiming to provide a measurement to estimate a disease’s relevance to immune responses.

4. Many chemokine/receptors are GPCRs so may be circular in your enrichment analysis.

Thanks. The associations that we predicted are between cytokines and non-cytokine genes. Then we found that many of the associations predicted for cytokines are receptors. For analysis, we only label these receptors (in graphs or tables) to show the percentages. No further analysis was applied to avoid confusion.
